# Postural control in healthy adults: Determinants of trunk sway assessed with a chest-worn accelerometer in 12 quiet standing tasks

**DOI:** 10.1371/journal.pone.0211051

**Published:** 2019-01-23

**Authors:** Fabienne Reynard, David Christe, Philippe Terrier

**Affiliations:** 1 Clinique romande de réadaptation, Sion, Switzerland; 2 Swiss federal institute of technology, Lausanne, Switzerland; Hochschule Trier, GERMANY

## Abstract

Many diseases and conditions decrease the ability to control balance. In clinical settings, there is therefore a major interest in the assessment of postural control. Trunk accelerometry is an easy, low-cost method used for balance testing and constitutes an alternative method to the posturography using force platforms. The objective was to assess the responsiveness of accelerometry in a battery of 12 quiet standing tasks. We evaluated the balance of 100 healthy adults with an accelerometer fixed onto the sternum. We used the average amplitude of acceleration as an indirect measure of postural sways. The tasks of increased difficulty were realized with or without vision. The battery of tasks was repeated four times on two different days to assess reliability. We analyzed the extent to which the task difficulty and the absence of vision affected the trunk sway. The influence of individual characteristics (age, height, mass, sex, and physical activity level) was also assessed. The reliability analysis revealed that four repetitions of the battery of tasks are needed to reach a high accuracy level (mean ICC = 0.85). The results showed that task difficulty had a very large effect on trunk sways and that the removal of vision further increased sways. Concerning the effects of individual characteristics, we observed that women tended to oscillate more than men did in tasks of low difficulty. Age and physical activity level also had significant effects, whereas height and mass did not. In conclusion, age, sex, and physical fitness are confounders that should be considered when assessing patients’ balance. A battery of simple postural tasks measured by upper-trunk accelerometry can be a useful method for simple balance evaluation in clinical settings.

## Introduction

Postural control is the act of maintaining, achieving or restoring a state of balance during any posture or activity [1]. Many diseases and conditions can alter postural control and, hence, increase the risk of falls [2–5]. Consequently, there is a major interest in postural control assessment for diagnostic purposes and for evaluation of the efficacy of therapies [2,3,5,6].

Dedicated neural circuits at the spinal and supraspinal levels are responsible for postural control [7]. Motor control centers receive inputs from visual, vestibular, and somatosensory systems. In turn, they control the muscles responsible for the postural response. Fundamentally, the task of postural control is to keep the body’s center of mass safely within the base of support [8]. Even during quiet, upright standing, the occurrence of small postural sways reflects the constant activity of postural control. When perturbations are minimal, ankle plantar flexors/dorsiflexors and invertors/evertors, are the major muscle groups responsible for postural response (ankle strategy) [8]. Regulation at hip level become more important in the case of stronger perturbations (hip strategy) [8]. More complex models of postural control suggest differential regulation in mediolateral (ML) and anteroposterior (AP) directions [9].

Clinical assessment of balance performance often requires distinguishing subtle changes in postural response that are not apparent during quiet upright standing. As a result, balance perturbation methods have been used to increase demand on postural control [10,11]. Postural control can be challenged through the shrinking of the base of support or through manipulations of sensory feedbacks. Specifically, a smaller base, as in tandem or one-leg stance, decreases stability [12,13]. Likewise, it is also possible to manipulate tactile and proprioceptive feedbacks from the feet with a compliant surface such as foam [10]. In addition, suppressing [14] or altering [15] visual feedbacks induces larger postural sways. In order to assess balance abilities, a combination of several standing tasks, challenging postural control in various ways, is therefore needed.

In addition to the effect of different types of balance task on postural response, idiosyncratic determinants play an important role in balance performance. In other words, individual characteristics can modify the ability to control postural sways. For example, children and older adults exhibit poorer postural control and larger postural sways [16,17]. In a similar way, several studies have also highlighted age-related changes in postural control throughout the life course [18,19]. Although several balance studies have suggested a difference between men and women [20,21], the topic remains controversial [18,19,22]. Excess body weight influences balance performance. Obese individuals therefore tend to exhibit larger postural sways [6,23]. Well-trained athletes have better postural control than average individuals [24]. Thus, it can be hypothesized that fit individuals, who are more physically active, may exhibit smaller postural sways. Regarding the clinical assessment of balance ability, it is crucial to characterize the determinants of postural control to clearly differentiate between pathological results and expected results given individual characteristics.

The golden standard to assess postural control is the force platform [25]. This consists of a device sensitive to forces applied on the ground by the subject. The center-of-pressure trajectory (postural sway) informs about the postural response. Classical methods—most often referred to as posturography—include the analysis of sway velocity (i.e., the speed at which the center-of-pressure moves) or the analysis of the spatial dispersion of the trajectories (area, root mean square (RMS), or length of path). Despite their accuracy, force platforms are cumbersome and limited to laboratory conditions. Accordingly, they cannot ensure a rapid postural evaluation in many settings, such as a practitioner’s office, a physiotherapist’s training session, or a patient’s home.

Wearable accelerometers have been suggested as an alternative method to evaluate postural control [26–28]. The rationale is to measure the acceleration of body segments induced by postural response. As a recapitulative index for postural corrective motions, the classical approach is to assess average dispersion of the acceleration signal over a given period of time [26,28,29]. With the advantage of low cost and portability, accelerometry is valid and reliable [3,5,28,30]. Accelerometry provides similar results as posturography with force platforms [31]. As a result, many successful applications of postural accelerometry have been reported. These include the following observations: trunk accelerometry reveals postural instability in Parkinson’s disease [3]; body sways measured with an accelerometer reflect athletic skill level [32]; and accelerometry can assess balance of diabetic patients with peripheral neuropathy [2].

Most accelerometer users attach the sensor to the lower back in order to measure the motions of the center of gravity [2,30,32,33]. However, some investigators have successfully assessed postural balance through upper-trunk accelerometry [6,34,35]. Indeed, measuring the acceleration of the upper trunk may not only offer better sensitivity to postural response but also yield specific information about trunk dynamics [36–38]. Data obtained by upper-trunk accelerometry are still sparse. For example, Dalton et al. showed that thorax acceleration can differentiate between premanifest and manifest Huntington’s disease subjects [34]. Although the methodology has been proven efficient, further studies are needed to confirm its responsiveness and clinical usefulness.

To further explore the efficacy and usability of accelerometry for clinical balance assessment, we designed a cross-sectional study to evaluate whether a battery of quiet standing tasks, instrumented with a chest-worn accelerometer, could assess postural control. We asked healthy adults to undergo increasingly difficult balance tasks that were known to increase postural response. In addition, we aimed to analyze how individual characteristics may interfere with postural control. In more details, we first evaluated the repeatability of the measurement. We then assessed whether acceleration amplitude could discriminate among the different balance tasks. Next, through specific recruitment, we focused on the effects of age and sex on postural response. Secondary determinants were leg dominance, body mass and height, and degree of sedentarity. We also assessed whether differences existed between the amplitude of AP and ML sways.

## Methods

### Participants

One-hundred healthy adults (50 males, 50 females), with no neurological or orthopedic conditions, participated in the study. We recruited ten males and females for each decade between 20 and 69 years of age. During the first visit, participants’ anthropometric characteristics were measured. Leg dominance was determined by asking the subjects which leg they would use to kick a ball [39]. We also asked them how many hours they spent exercising per week. Table 1 summarizes the participants’ characteristics.

**Table 1:** Characteristics of the participants.

	Total (N = 100)	20–29yr (N = 20)	30–39yr (N = 20)	40–49yr (N = 20)	50–59yr (N = 20)	60–69yr (N = 20)
Age (year)	44.2	24.7	34.6	43.9	54.8	63.3
	(14.1)	(2.8)	(2.8)	(2.9)	(2.7)	(3.2)
Body mass (kg)	70.2	68.4	65.4	74.2	71.1	72.0
	(14.6)	(11.9)	(12.8)	(15.6)	(14.4)	(17.2)
Body height (m)	1.72	1.74	1.70	1.74	1.71	1.69
	(0.07)	(0.06)	(0.08)	(0.06)	(0.08)	(0.06)
Exercise (hours/week)	3.0	3.8	3.4	2.3	2.6	2.9
	(2.6)	(3.5)	(2.6)	(2.3)	(1.6)	(2.3)

Values are means (standard deviations)

The study was carried out in accordance with the principles enunciated in the current version of the Declaration of Helsinki and the requirements of Swiss law and the Swiss regulatory authority. Ethical approval was obtained from the Commission Cantonale Valaisanne d’Ethique Médicale (Sion, Switzerland) prior to the study (protocol ID: CCVEM 019/11). The participants were informed of procedures, risks and benefits, confidentiality, and the voluntary nature of participation, before signing a consent form.

### Data collection

#### Instrument

The participants wore a tri-axial accelerometer (Physilog system, GaitUp, Lausanne, Switzerland; sampling rate 200Hz, 16-bit resolution [2]). This instrument has already been successfully used by others to assess standing balance [2]. It was fixed with a belt to the anterior upper-trunk level, 5 cm under the sternal notch. The accelerometer measured trunk accelerations in earth acceleration units (g) along ML, vertical, and AP axes. The sensor was connected by means of a 1.5 m cable to the data logger held by the experimenter, who manually switched on the recording at the onset of each standing task and switched it off just after.

#### Measurements

The participants stood barefoot to ensure footwear had no effect on postural control. They had to perform 12 tasks in a different randomized order for each repetition. Each task lasted 30 s, as recommended for postural stability assessment [40]. Table 2 describes the assigned tasks, along with the abbreviations used to describe them hereafter. At the onset of each task, participants were asked to keep the arms alongside the body and to maintain a light knee flexion (approximately 5°), with the purpose to avoid knee extension locking. During the tasks, they had to stand as still as possible, gazing straight ahead. In the case of a large imbalance, a crossbar was available in front of them to catch themselves before falling; in this case, they were asked to hold the crossbar as briefly as possible and to resume the task as soon as possible. Twenty seconds of rest was allowed between tasks. The whole procedure was repeated a couple of minutes later. A week later (mean: 9.1 days, SD: 3.6), an identical measurement session took place.

**Table 2:** Description of the standing tasks.

Code	Name	Description
FA_EO	Feet apart, eyes open	standing with feet 10 cm apart and externally rotated at 10° with eyes open
FA_EC	Feet apart, eyes closed	standing with feet 10 cm apart and externally rotated at 10° with eyes closed
FT_EO	Feet together, eyes open	standing with feet together with eyes open
FT_EC	Feet together, eyes closed	standing with feet together with eyes closed
FF_EO	Feet together on foam, eyes open	standing with feet together on medium-density foam with eyes open
FF_EC	Feet together on foam, eyes closed	standing with feet together on medium-density foam with eyes closed
OD_EO	One leg, dominant limb, eyes open	standing on the dominant limb with eyes open
OD_EC	One leg, dominant limb, eyes closed	standing on the dominant limb with eyes closed
ON_EO	One leg, non-dominant limb, eyes open	standing on the non-dominant limb with eyes open
ON_EC	One leg, non-dominant limb, eyes closed	standing on the non-dominant limb with eyes closed
BD_EO	One leg, board, dominant limb, eyes open	standing on the dominant limb on a rocking board, unstable in the frontal plane, with eyes open
BN_EO	One leg, board, non-dominant limb, eyes open	standing on the non-dominant limb on a rocking board, unstable in the frontal plane, with eyes open
OA_EO	One leg, average, eyes open	Average of OD_EO and ON_EO
OA_EC	One leg, average, eyes closed	Average of OD_EC and ON_EC
BA_EO	One leg, board, average, eyes open	Average of BD_EO and BN_EO

### Data processing

After downloading acceleration data to a computer, the raw signals were exported to Matlab (Mathworks, Natick, MA). Firstly, the 3D signals were reoriented taking advantage of the capacity of accelerometers to function as inclinometers [28]. This procedure attenuated the effects of sensor orientation, which might not be perfectly constant among participants. The vertical signal was not analyzed as postural sway occurs in the transverse plane. Secondly, the acceleration signals (AP and ML) were low-pass filtered to remove high frequency noise. There is no clear consensus about the optimal filtering for postural sway assessment with accelerometers. Most of the postural oscillations occurs at low frequency [26]. Upper bandwidth limits used in accelerometer studies are: 3.5Hz [3], 11Hz [35], 20Hz [26,32], 50Hz [29], 100Hz [5], and 250Hz [34]. Based on this literature review, and because we observed that >95% of the signal power was below 15Hz, we used a cutoff at 30Hz (12th order Butterworth).

We kept a constant number of 6000 samples (each 30 seconds in duration) across the signals. Finally, as a measure of sway amplitude, the RMS of the acceleration was computed over the 30-second signals. RMS is the standard method used in most accelerometry studies [2,3,5,26,27,29,31–34].

### Data analysis

#### Repeatability

The within- and between-session repeatability were analyzed to assess the number of tests that were needed to reach a sufficient degree of reliability [41,42]. For brevity’s sake, the detailed methodology and results are available in the supplemental material (S1 File). Here, we only present an estimate of the repeatability of four measurements. The Spearman-Brown prophecy formula [43] was applied on the repeatability results regarding one repetition (see S1 File) to extrapolate the expected repeatability when four repetitions were averaged together.

#### Descriptive and inferential statistics

**Fig 1:**
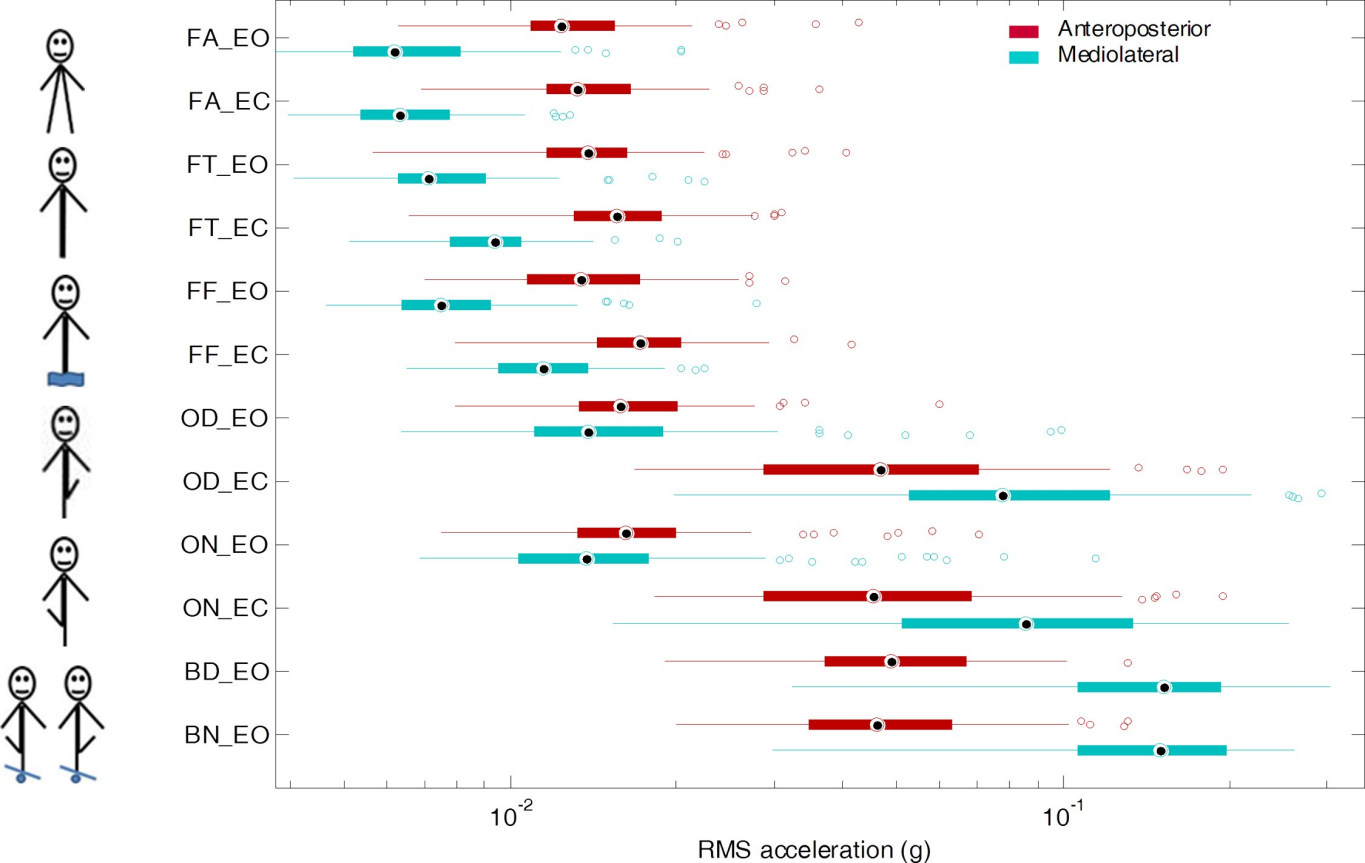
Descriptive statistics of sway amplitude. One-hundred participants performed twelve 30-s standing tasks (see Table 2 for tasks description). Trunk sways were measured with an accelerometer. The root mean square (RMS) of the 30-s acceleration signals assessed the average amplitude of the sways. Boxplots show medians, quartiles, data extents, and outliers, separately for the AP and mediolateral axis. A logarithmic scale was used to enhance the visualization.

We used standard boxplots—including median, quartiles, extent of the data and outliers—to graphically depict the individual results, averaged over the four repetitions (Fig 1). Due to the large spread between tasks and individuals, we improved visualization by means of a logarithmic scale. In addition, Table 3 displays the medians and interquartile ranges (IQR). To ease the reading of the table, we multiplied raw RMS values by 1000, converting g units to mg. Histograms showing the distribution of the sway amplitude for each standing task can be found in the supporting information (Figure A in S2 File). Supporting information also contains scatter plots that display bivariate distributions of sway amplitudes against other variables across standing tasks (Figures B–S in S2 File).

**Table 3:** Repeatability (ICC) of the postural control measures.

	Anteroposterior			Mediolateral		
	ICC	CI		ICC	CI	
FA_EO	0.81	(0.74	– 0.86)	0.83	(0.77	– 0.88)
FA_EC	0.86	(0.80	– 0.90)	0.77	(0.68	– 0.83)
FT_EO	0.77	(0.67	– 0.83)	0.87	(0.82	– 0.91)
FT_EC	0.84	(0.78	– 0.88)	0.85	(0.79	– 0.89)
FF_EO	0.71	(0.60	– 0.79)	0.79	(0.70	– 0.85)
FF_EC	0.77	(0.67	– 0.84)	0.75	(0.67	– 0.82)
OD_EO	0.84	(0.78	– 0.89)	0.82	(0.75	– 0.87)
OD_EC	0.88	(0.84	– 0.92)	0.93	(0.90	– 0.95)
ON_EO	0.73	(0.62	– 0.80)	0.88	(0.83	– 0.91)
ON_EC	0.89	(0.85	– 0.92)	0.92	(0.89	– 0.94)
BD_EO	0.84	(0.77	– 0.88)	0.90	(0.86	– 0.93)
BN_EO	0.87	(0.82	– 0.91)	0.90	(0.86	– 0.93)
OA_EO	0.84	(0.78	– 0.89)	0.91	(0.87	– 0.93)
OA_EC	0.94	(0.91	– 0.96)	0.95	(0.94	– 0.97)
BA_EO	0.91	(0.87	– 0.93)	0.93	(0.91	– 0.95)

ICC: intraclass correlation coefficient. CI: confidence interaval. See Table 2 for the other abbreviations

Because the RMS acceleration is strictly positive, data were expected to be not normally distributed (right skewed, Figure A in S2 File). We applied therefore Wilcoxon’s signed rank tests for the null hypothesis that the difference between conditions comes from a distribution with a zero median (paired test). We used Bonferroni’s correction to mitigate the multiple-comparison bias. We compared axes (AP vs. ML), vision conditions (EO vs. EC.) and leg dominance (dominant vs. non-dominant) across standing tasks (see Table 3 and the result section).

#### Multivariate adaptive regression splines (MARS)

We sought to evaluate potential associations between participants’ characteristics and sway amplitude. To this end, we selected the multiple adaptive regression splines (MARS) algorithm because of its appropriateness to handle nonlinear relationships, its ability to detect interactions among predictors, and its good bias-variance tradeoff [44]. As a nonparametric regression method, MARS offers a very good alternative to multiple regression analysis when many predictors and interactions need to be assessed [45]. Indeed, MARS can include important variables in the model and excludes unimportant ones (automatic variable selection). In short, the MARS algorithm combines the logic of stepwise multiple regression and decision trees. Piecewise linear splines (basis functions) split predictors around knots. A forward pass recursively adds basis functions that reduce the residual error. Then, a backward phase prunes the model by penalizing model complexity. For further information, refer to the tutorial article of Everingham and Sexton [46].

We implemented MARS through the Matlab package ARESLab [47]. We applied piecewise linear modelling (‘cubic’, false). We tuned the model to include potential pairwise interactions (‘maxInteraction’, 2). The number of knots for building basis functions was limited by imposing a minimum of eight points between knots (‘UseMinSpan’, 8). In the same way, to lower spurious fitting at the end of data intervals, we set a minimum span of 15 near the ends (‘useEndSpan’, 15). Other tuning parameters were set to default values. The predictors were age, sex, body height, body mass, and exercise. The dependent variables were the sway amplitudes (RMS). To attenuate potential bias induced by outliers, we excluded values that were more than 2.5 standard deviations away from the mean. Detailed MARS results are presented in S2 File (fitting curves). 3D plots were used to illustrate relevant interactions among predictors.

## Results

We planned to measure 4800 standing tasks, that is, 12 tasks within two intrasession repetitions and two intersession repetitions measured in 100 subjects. Although there were 149 missing tasks (3%) due to organizational and technical issues, we obtained at least two measures per participant for each standing task. No participants experienced very large imbalance and touched the safety bar longer than a couple of seconds. No participants reported fatigue and lack of attention during the experiment.

### Repeatability

The repeatability analysis (see S1 File) demonstrated a low repeatability when a single repetition was considered. Repetitions had to be averaged together to obtain ICCs exceeded the 0.7 threshold commonly admitted as a lower limit for acceptable reliability [48]. Table 3 shows the extrapolated reliability of four measurements of standing tasks. The average ICC was 0.83 in the AP direction (range 0.71–0.94), and 0.87 in the ML direction (range 0.75–0.95).

### Effect of task difficulty

The demanding tasks were logically associated with larger sways (Fig 1 and Table 4). Indeed, the sway amplitudes rose from 0.0062 g for standing feet apart up to 0.15 g when standing on the rocking board (i.e., a 24-fold increase). In more details, in EO conditions, standing with feet together increased sway amplitudes by 11% along the AP axis, and by 15% along the ML axis as compared to standing with feet apart. Standing on foam increased AP sways by 8% in the AP direction, and by 21% in the ML direction as compared to standing with feet apart. Regarding the one-leg tasks, considerable between-subject variance indicates very variable mastering among the participants.

**Table 4:** Descriptive statistics and comparisons between conditions.

N = 100		FA EO	FA EC	FT EO	FT EC	FF EO	FF EC	OD EO	OD EC	ON EO	ON EC	BD OD	BN EO
Descriptive stats	AP	12.4	13.2	13.8	15.6	13.4	17.2	15.8	46.6	16.2	45.3	48.9	46.2
		(4.6)	(5.0)	(4.7)	(5.8)	(6.4)	(6.1)	(6.7)	(41.7)	(6.7)	(39.7)	(29.9)	(28.4)
	ML	6.2	6.3	7.1	9.4	7.1	9.4	7.5	11.5	13.8	85.3	152.2	149.6
		(2.9)	(2.4)	(2.8)	(2.7)	(2.8)	(2.7)	(2.9)	(4.3)	(7.5)	(83.0)	(86.7)	(90.6)
Axes: AP vs.ML		**-50%**	**-52%**	**-49%**	**-40%**	**-44%**	**-33%**	-13%	**67%**	**-15%**	**89%**	**211%**	**224%**
Vision: EC vs. EO	AP	**7%**		**13%**		**28%**		**195%**		**180%**		N/A	
	ML	2%		**33%**		**54%**		**463%**		**520%**		N/A	

Top rows show the descriptive statistics via medians and interquartile ranges across standing tasks. Unit is mg (g = earth acceleration unit). Below, relative differences between conditions and inferential tests (Wilcoxon rank tests) are presented. Significant results are in bold print. Significance thresholds (Bonferroni-corrected for multiple comparisons) are: p = 0.004 for axes (12 comparisons); and p = 0.01 for vision (5 comparisons). ML: mediolateral. AP: anteroposterior. EC: eyes closed. EO: eyes open. For other abbreviations, see Table 2.

### Directional effect

Sway amplitudes differed between the AP and ML axes (Fig 1 and Table 4): regarding bipedal tasks (i.e., FA, FT, and FF) the trunk oscillated more in the sagittal plane (AP) than in the frontal plane (ML). Conversely, the rocking-board tasks (BD and BN) induced a stronger sway in the ML direction as compared to the AP direction. The one-leg tasks (OD and ON) exhibit intermediate results: with eyes open, the differences between both axes are limited, but, with eyes closed, the ML sways were stronger than the AP sways.

### Importance of vision

The results show a strong dependence on visual inputs to control sway amplitude (Fig 1 and Table 4). In addition, visual deprivation effects rise sharply with task difficulty. Whereas no significant difference was indeed noted in the FA task, a prominent relative difference of about 500% was evident in the one-leg tasks (ON and OD). Besides, the results show a directional dependence: in the absence of visual information, trunk oscillations increased more in the ML than in the AP direction.

### Effect of leg dominance

In the one-leg tasks, the results show no leg dominance effect. Indeed, postural sway is of a similar amplitude whatever leg was used for performing the task. In more detail, with eyes open (ON and OD), a non-significant difference of 7% and 4% was observed in AP and ML directions (AP: *p* = 0.42; ML: *p* = 0.89, respectively). Similarly, with eyes closed, the relative differences were AP: 2% (*p =* 0.50); ML: 6% (*p* = 0.10). Concerning the rocking-board task, the differences were AP: 3% (*p =* 0.12); ML: 1% (*p =* 0.69). Accordingly, averaging the results of both legs seems valid.

### Idiosyncratic determinants of sway amplitude

Across the bipedal tasks (FA, FT, and FF), the MARS results show associations between postural sway and subject characteristics only for the ML direction. Accordingly, Table 5 shows only ML results for those tasks. Conversely, across unipedal tasks (OA and BA) the MARS results show relevant associations for both AP and ML axes.

**Table 5:** Idiosyncratic determinants of sway amplitude.

		R^2^	MARS Model
FA_EO	ML	0.13	BF1 = max(0, **AGE**-40), y = 6.09 + 0.09*BF1
FA_EC	ML	0.22	BF1 = max(0, **AGE**-32), BF2 = max(0, 1-**SEX**), BF3 = BF2 * max(0, 27-**AGE**) y = 6.49 + 0.04*BF1–1.03*BF2 + 0.38*BF3
FT_EO	ML	0.25	BF1 = max(0, 42-**AGE**), BF2 = max(0, 1-**SEX**), BF3 = max(0, 58-**AGE**) y = 9.34 + 0.21*BF1–0.96*BF2–0.16*BF3
FT EC	ML	0.22	BF1 = max(0, **AGE**-28), BF2 = max(0, 28-**AGE**), BF3 = max(0, **SEX**), BF4 = max(0, **HEI**-1.77) * max(0, **AGE** -43) y = 7.34 + 0.06*BF1 + 0.29*BF2 + 1.57*BF3 + 3.57*BF4
FF EO	ML	0.10	BF1 = max(0, **SEX**), BF2 = max(0, **HEI** -1.67) * max(0, **AGE** -60) y = 7.09 + 1.51*BF1 + 8.98*BF2
FF_EC	ML	0.26	BF1 = max(0, **AGE**-28), BF2 = max(0, 28-**SEX**), BF3 = max(0, **SEX**) y = 8.23 + 0.13*BF1 + 0.74*BF2 + 1.62*BF3
OA EO	AP	0.21	BF1 = max(0, **AGE**-40) * max(0, 3.5-**EXE**) y = 16.11 + 0.08*BF1
OA_EO	ML	0.36	BF1 = max(0, **AGE**-27)*max(0, **MAS**-67), BF2 = max(0, **AGE** -27)*max(0, **EXE**-3.5), BF3 = max(0, **MAS**-82), BF4 = BF3 * max(0, 35 -AGE), BF5 = max(0, 82-**MAS**) * max(0, **AGE**-27), BF6 = max(0, 82-**MAS**) * max(0, 27-**AGE**) y = 10.05 + 0.04*BF1–0.07*BF2–1.03*BF3 + 0.15*BF4 + 0.01*BF5 + 0.06*BF6
OA_EC	AP	0.33	BF1 = max(0, **AGE**-32), BF2 = BF1 * max(0, **EXE**-3), BF3 = max(0, 1.75-**HEI**), BF4 = max(0, 65-**MAS**) * max(0, 32-**AGE**) y = 37.37 + 1.47*BF1–0.25*BF2–110.20*BF3 + 0.46*BF4
OA_EC	ML	0.27	BF1 = max(0, 33-**AGE**), BF2 = max(0, 46-**AGE**) y = 108.31 + 10.73*BF1–5.49*BF2
BA_EO	AP	0.22	BF1 = max(0, **AGE**-29), BF2 = max(0, 29-**AGE**) y = 36.05 + 0.83*BF1 + 2.38*BF2
BA_EO	ML	0.14	BF1 = max(0, 29-**AGE**), BF2 = max(0, **AGE**-29) * max(0, **HEI**-1.79) y = 139.65 + 7.67*BF1 + 70.05*BF2

Results of the MARS models. Sway amplitude (RMS) for each standing task is the dependent variable (y). Predictors are age (AGE), sex (SEX), height, (HEI), mass (MAS), and exercise (EXE). BF: basis function. AP: anteroposterior. ML: mediolateral. EC: eyes closed. EO: eyes open. For other abbreviations, see Table 2.

Regarding bipedal tasks, individual characteristics explained 20% of the variance of trunk sways (Table 5, R^2^ range: 0.10–0.26). Unipedal tasks are slightly more discriminant: MARS analyzes revealed that on average 26% of the sway variance among participants were explained by the predictors (Table 5, R^2^ range: 0.14–0.36).

#### Age

Age explains the most variance among the participants (Table 5). Indeed, age was associated as the main effect with sway amplitudes in seven out of nine of the standing tasks. Age also interacts with other predictors, especially in one-leg tasks (OA and BA). Large sway amplitudes tend to be more frequent in older individuals (Figures M, O, P, R in S2 File). In addition, a J-shaped relationship—with the lowest sway amplitude at around 30 years of age—was observed in several tasks (FA_EO; FT_EO; FF_EO; OA_EO; BA_EO, Figures M, O, R in S2 File).

#### Sex

An effect of sex is evident in all the bipedal tasks (FA, FT, and FF). Women tended to exhibit larger sways along the ML axis than men.

#### Height, mass, and exercise

**Fig 2:**
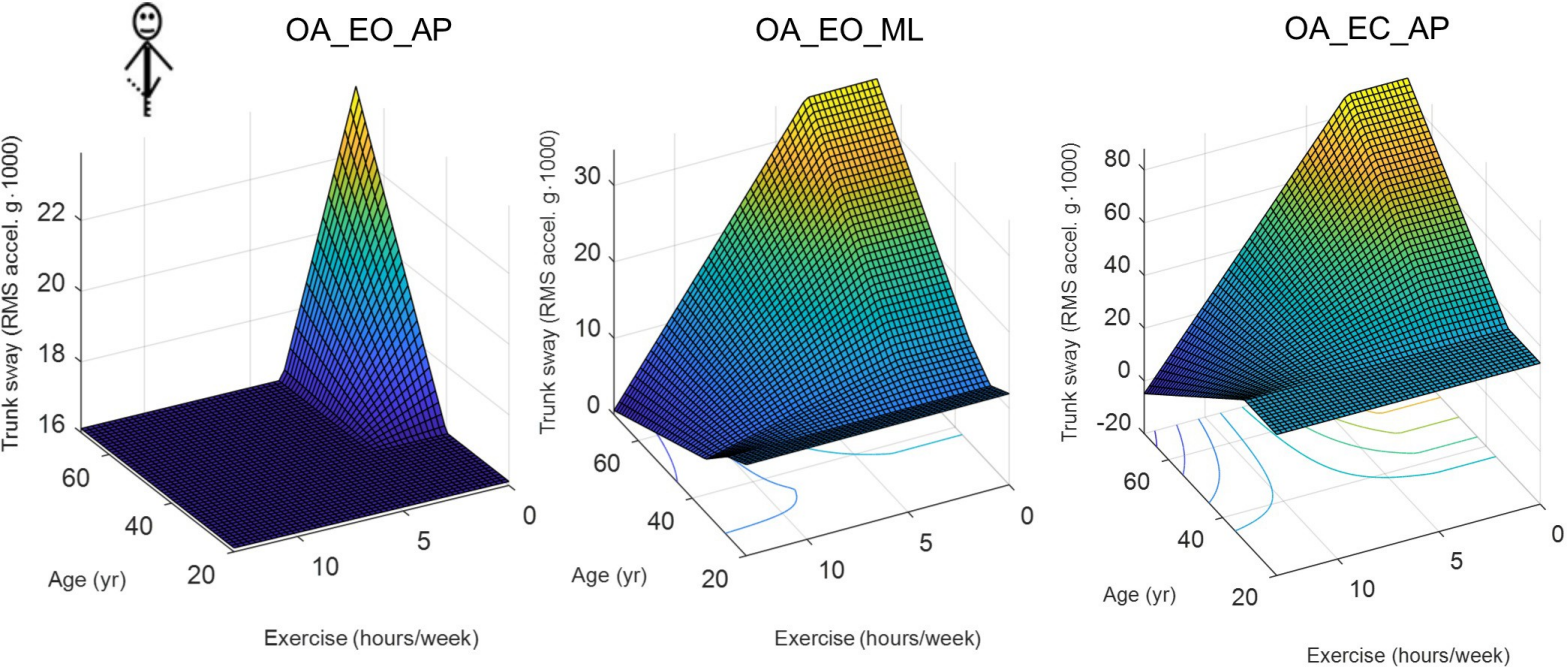
Age-by-exercise interactions. 3D plots of the MARS model output for the one-leg standing tasks (see Table 2 for the exact description of the tasks). The equations of MARS models are in Table 5.

Finally, other factors are associated inconsistently with postural sway mostly through interactions with age, especially in the more difficult tasks. A closer examination of age-by-exercise interactions in 3D plots reveals a kind of protective effect of exercise against aging for more demanding tasks (Fig 2). Thus, older participants exhibit a higher postural sway only if they report fewer hours of exercise per week.

## Discussion

We measured 100 healthy adults performing 12 quiet standing tasks with an accelerometer fixed onto the sternum. We used the average amplitude of the acceleration signal (RMS) as an indirect measure of the thorax sway, and, hence, as an index of postural response. We aimed at highlighting typical traits of postural control. We noticed low repeatability of a single sway measure (S1 File), which emphasizes the need for averaging several trials together. We confirmed three well-known observations: 1) sways increase with task difficulty; 2) vision is important to attenuate postural sways; and 3) sways increase with age. In addition, we highlighted more subtle facts: 1) postural control acts differently along the AP and ML directions; 2) leg dominance has no effect on sway amplitude; 3) women oscillate laterally slightly more than men when performing bipedal standing tasks; 4) body mass and height do not have a relevant effect on sway amplitude; and 5) being more active may protect against age effects.

It is well established that repeated measures are needed to obtain a reliable estimation of postural control. In a study that measured acceleration RMS at low-back level, the repeatability of a single measure was low (ICC in bipedal tasks: 0.22–0.57) [49]. Similarly, it has been reported [50] that the RMS of the center-of-pressure (COP) trajectory measured during 30 seconds through force platforms also exhibits low intrasession reliability (ICC 0.35–0.39). Therefore, the low reliability is not essentially due to measurement methods, but mostly due to the inherent variability of postural control. A higher repeatability has been reported for unipedal tasks (ICC: 0.69–0.85), very likely due to the higher inter-individual variability observed for these challenging tasks [30]. Our results regarding the within-session repeatability of one measurement (see S1 File) are in line with these studies (bipedal ICCs: 0.38–0.63, unipedal ICCs: 0.40–0.83). Several authors recommend averaging several tests together to increase the reliability of balance assessment [21,50,51]. Others recommend assessing balance on different days to establish a valid baseline [26]. Our analysis supports these recommendations (Table 3): the reliability of the average of four tests collected on two different days were indeed high (average ICC: 0.85).

Because leg dominance seems to have no relevant effect according our results and the literature [39], averaging the results from both legs could also be a solution to improve reliability. However, many pathologies and conditions can induce left-right differences between lower limbs. If anything, comparing results from both legs may be informative.

Previous studies have used varying standing tasks to investigate aspects of postural control [9,10,13,27,52]. The rationale is to challenge somatosensory, visual, and vestibular inputs differentially. Our choice of standing tasks (Table 2) accounts for this diversity. The results indicate that measuring trunk acceleration at chest level can discriminate between the chosen tasks. As expected, RMS values do indeed increase with task difficulty (Fig 1 and Table 4). In our sample of healthy individuals, the increase in postural sway attested by higher RMS values is limited among bipedal tasks (+11%–+21%), which reflects the resilience of postural control. It is likely that vision and vestibular input compensate for perturbed somatosensory inputs. In contrast, one-leg tasks, especially when standing on the rocking platform, induced larger postural sways in line with the challenge demanded by those tasks.

In bipedal tasks, the results (Table 4) show that sway amplitude is greater in the sagittal plane than in the frontal plane (relative difference: 44% to 52%). Two studies that measured RMS acceleration at the low-back level during quiet standing have reported similar results [2,49]. Corrections at ankle joint level may dominate postural control along the AP axis, whereas the hip joint could be more important along the ML axis [9]. The morphology of the ankle joint allows more freedom longitudinally than laterally: this is probably why the amplitude of movements and the force needed to control them are larger in the sagittal plane. In contrast, the results show fewer differences between axes in the one-leg tasks carried out with eyes open (13% to 15%). Here, too, comparable results have been found [49]. Probably, standing on one leg requires more work from hip abductors/adductors to shift the center of mass laterally on the weight-bearing leg, which increases sways in the ML direction.

Postural control relies on sensory inputs in a complex way [53]. It is well established that low-frequency sways are preferentially stabilized by vision [15,54]. In quiet standing, postural control can compensate for vision deficits using other sensory inputs [53,55,56]. In confirmation of this, we observed that visual deprivation has no substantial effect when standing feet apart (Table 4). In more challenging situations, the literature reports that postural control relies preferentially on vision, such as in reduced stance-width tasks [12] or in one-leg standing [14]. Similarly, our results confirm that the more difficult the standing task, the more the sways increase with eyes closed (Table 4 and Fig 1).

In eyes-closed conditions, the results show that postural sways increase more in the ML direction than in the AP direction (Table 4). The effect is especially strong in one-foot standing. We hypothesize that the tilt of landmarks in the visual field induced by the left-right head movements constitutes relevant cues that postural control can exploit to correct ML sways. In contrast, forward/backward motions induce fewer changes in the visual field, which might explain why somatosensory and vestibular inputs are more likely to correct AP sways.

The results suggest that older adults exhibit larger postural sways. In fact, in contrast to the other predictors (sex, age, body mass and height, and exercise), age was selected by the MARS algorithm for all the standing tasks (Table 5). Furthermore, a close inspection of the MARS equations (Table 5) reveals nonlinear associations. An inflexion point between 30 and 40 years of age is thus most often observed. Many other studies have found that postural sway increases in older people (>65 year old) [16,57–59]. In addition, similar to our results, other studies have shown that middle-aged adults show a deterioration of balance function [18,19]. One explanation could be that muscle strength declines at a higher rate after the fourth decade of life [60].

We also observed that younger participants (under 25 years of age) might also have larger postural sways (J-shaped curve, see, e.g., Figure R in S2 File). Likewise, a U-shaped relationship has been observed between age and postural sway speed, with higher values in children and older adults [17]. The maturation of an optimal postural control might need the accumulation of situational experiences that is only reached in the first decade of adulthood [17].

The MARS results (Table 5) revealed that women tended to oscillate more than men along the ML axis during bipedal tasks. Although significant, the effect was moderate (standardized effect size about 0.5). Several have shown a gender effect through the analysis of the COP trajectories during quiet standing [20–22]. Others, however, did not find any relevant gender effect on postural control [39,57], report opposite results [18], or are not conclusive [19]. Body mass and height were not retained by most of the MARS analyses; thus, it is unlikely that the sex effect is due to anthropometric differences between male and female. Considering that the hip joint is particularly important for ML balance in quiet standing [8], mechanisms explaining this sex difference might imply strength and control of abductors/adductors of the hip, and pelvic anatomy. Besides, Ku and colleagues suggest that the larger Q-angle found in women may increase the ML sway [22]. Other sources also highlight sex differences in hip abductor muscle properties [61], in hip abductor function when landing from a jump [62,63], in hip adduction and internal rotation during sport maneuvers [64], and in anterior pelvic tilt and thigh internal rotation [65]. Further studies are needed to investigate these points in the context of postural control.

The inconsistent associations of anthropometric variables with sway amplitude (Table 5) suggest that lighter/heavier or smaller/taller persons do not tend to have a different postural control than the average individual. However, note that the distribution of the anthropometric variables in our sample reflects the normal distribution in the population, in which extreme values are logically underrepresented. Accordingly, obese individuals may exhibit altered postural control, as pointed out by other studies with dedicated designs [6,23].

We postulated that more active individuals might exhibit better postural control through regular practice of exercise. In support of this hypothesis, other researchers have observed that strength training can improve balance ability in the elderly [66,67]. However, our results support this hypothesis only partially. On the one hand, age-by-exercise interactions highlight that older participants maintained low postural sways if they were more active (Fig 2). On the other hand, however, the skewed distribution of exercise levels among the participants (see Figure B-S in S2 File) might have favored spurious correlations. Studies specifically designed for detecting the exercise effect on postural control are needed to clarify the issue further.

The study has two limitation: first, we did not compare thorax accelerometry with conventional accelerometry that measures trunk acceleration at the lower back level. Although our results tend to indicate that a chest-worn accelerometer is responsive enough to assess postural control, further studies are needed to directly compare the performance of both upper-trunk and lower-trunk accelerometry. Second, given the upper-trunk placement of the accelerometer, the respiratory movements were recorded in addition to the postural sways. This might have influenced the results of the tasks that induced minimal sways (FA, FT). Because postural control compensates for respiration [68], it is difficult to distinguish respiratory movements from the postural corrections induced by them. This requires further investigations.

## Conclusions

The current study shows that a battery of standing tasks measured with a wearable accelerometer fixed to the upper trunk can evaluate postural control thoroughly. Whereas most other studies have focused on specific aspects of balance, our study covers a large spectrum of explanatory variables. The method seems responsive enough to distinguish among those diverse effectors and offers comparable reliability as other methods.

Nowadays, accelerometers are cheap, and readily available sensors are embedded in many devices, such as smartphones and activity trackers. Accelerometers are easy to use for the practitioner and non-invasive for the patient. Furthermore, the computation of acceleration amplitude through RMS is straightforward and does not require specialized software. Therefore, in many settings, we think that wearable sensors sensitive to movement could replace classical posturography using force platforms with positive effect.

From a physiological perspective, our results show that subtle variations of postural sways can reveal underlying individual characteristics. Notably, we detected a gender effect that has only been inconsistently observed so far. Likewise, we observed a mitigating effect of exercise that has not been reported so far in middle-aged adults and that deserves further studies.

From a clinical perspective, our findings identify which confounders should be considered when assessing patients’ balance. Prominently, postural outcomes must be adjusted for age, even in young and middle-aged adults. In addition, adjustments for sex should be realized in case of bipedal standing tasks. The RMS results (Table 4) can serve as reference values, and MARS equations (Table 5) can be used to adjust values according to individual characteristics. However, further investigation is required to tailor the balance tasks to the needs and capabilities of specific patients, to establish reference values for different pathologies, and to explore the method’s sensitivity to change.

## Supporting information

S1 File Repeatability analysis. The document contains a detailed analysis of the intra- and inter-session repeatability of thorax sway measures.
(PDF)

S2 File Supplementary figures. The document contains supplementary figures: histograms, scatter plots, and fitted curves of the MARS analysis.
(PDF)

S3 File Spreadsheet of raw data. (XLSX)
