# Supplementary material for: Postural control in healthy adults: Determinants of trunk sway assessed with a chest-worn accelerometer in 12 quiet standing tasks
Source: PLoS One. 2019 Jan 23;14(1):e0211051. doi: 10.1371/journal.pone.0211051 (PMC6344019; doi:10.1371/journal.pone.0211051)
Supplement: S1 File — The document contains a detailed analysis of the intra- and inter-session repeatability of thorax sway measures. (PDF) [file pone.0211051.s001.pdf]

Postural control in healthy adults: determinants of trunk sway assessed with a chest-worn accelerometer in 12 quiet standing tasks

FABIENNE REYNARD, DAVID CHRISTE, PHILIPPE TERRIER

Repeatability analysis

Methods

To evaluate the repeatability (R) of the trunk sway measurements, we applied a method based on linear mixed-effects models (LMM) [1]. The principle is identical to the classical ANOVA approach, which is used for intraclass correlation coefficients (ICC) [2]: it consists of computing the variance ratios that indicate how much variance can be attributed to the between-subject variance as opposed to the total variance. The advantage of LMM is its ability to analyze unbalanced datasets (i.e., different numbers of repetitions across subjects).

We used the R package rptR [3]. We applied Gaussian models (‘rptGaussian’) that were estimated through restricted maximum likelihood (REML). The 95% confidence intervals were computed by bootstrapping (‘nboot = 1000’). Two approaches were used: 1) we estimated R from all the available data, which assessed the reliability of one sway measurement; and 2) we averaged the data from each session separately before computing R, which assessed the intersession (week-to-week) repeatability.

Results

Overall (Fig. S1A) reliability is low. Most of the tasks did not reach the 0.7 threshold, which is the lower limit commonly required for acceptable reliability [4]. Seven R-values are even below the 0.5 threshold, which means that within-subjects variance exceeds between-subjects variance. Logically, the averaged one-leg tasks (OA and BA) exhibit the best reliability. With regard to intersession reliability (Fig. S1B), greater uncertainty is observed (larger confidence intervals). The results for bipedal tasks (FA, FT, and FF) are comparable to the overall results. On the other hand, one-leg tasks exhibit higher R-values and satisfactory reliability levels, with the exception of ON_EO_ML.

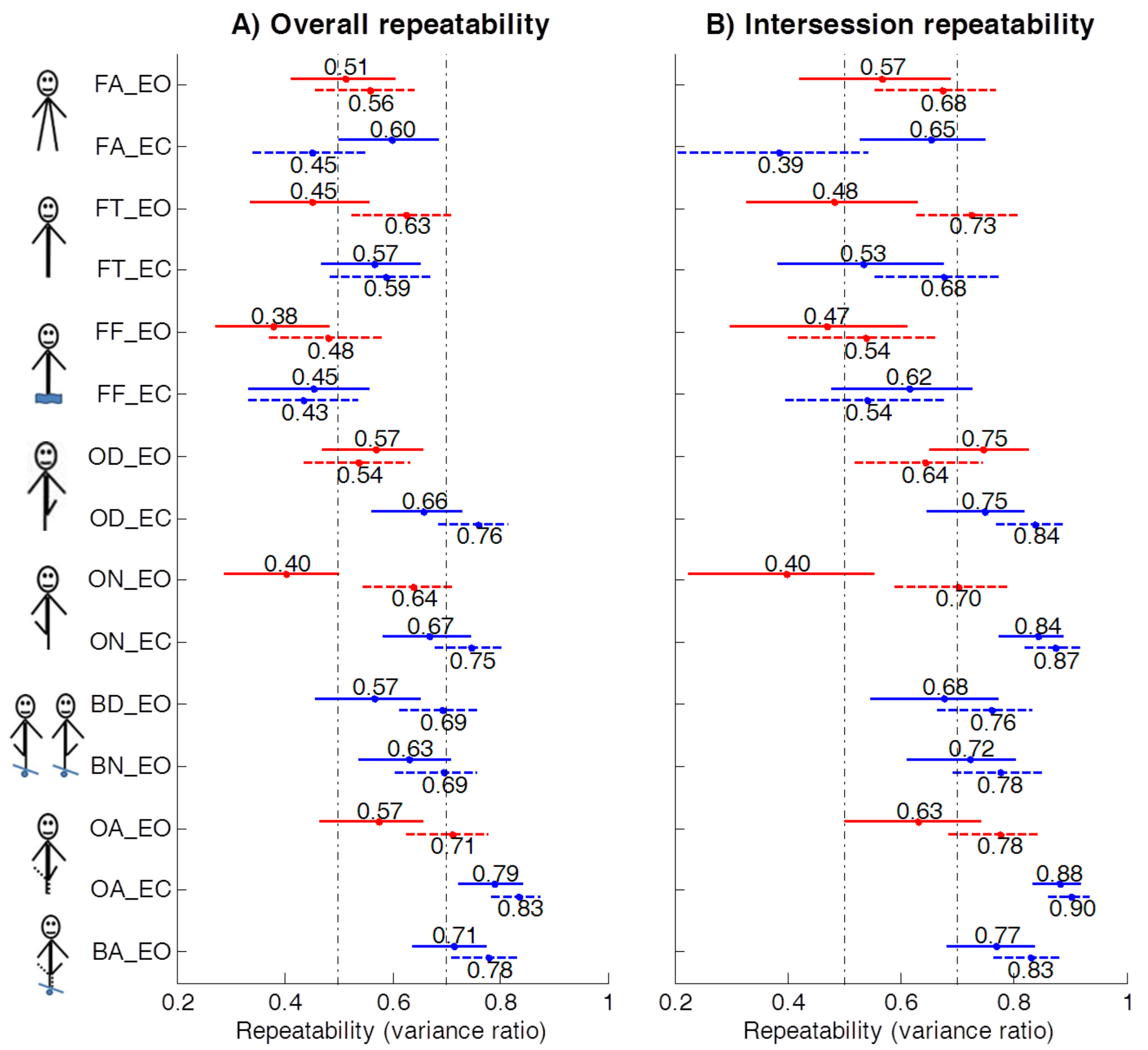

Figure S1. *Repeatability (ICC) of the trunk sway measures. Variance ratios (i.e. between-subjects variance to total variance) were estimated through linear mixed-effects modelling for each standing task (see table 1 in the main document for tasks description). Overall repeatability is the repeatability for a single measure of the task. Intersession repeatability is the repeatability when the measures in each session are averaged (week-to-week repeatability). Lines are 95% confidence intervals computed by bootstrapping. Red: tasks realized with eyes open. Blue: tasks realized with eyes closed. Continuous lines: trunk sway amplitudes in the AP direction. Discontinuous lines: trunk sway amplitudes in the mediolateral direction. Vertical dashed lines are drawn at ICC = 0.5 (equality between within-subject and between-subject variance) and ICC = 0.7 (minimal value commonly admitted for acceptable reliability).*

References

1. Nakagawa S, Schielzeth H. Repeatability for Gaussian and non-Gaussian data: a practical guide for biologists. Biol Rev Camb Philos Soc. 2010;85: 935–956. doi:10.1111/j.1469-185X.2010.00141.x

2. Weir JP. Quantifying test-retest reliability using the intraclass correlation coefficient and the SEM. J Strength Cond Res. 2005;19: 231–240. doi:10.1519/15184.1

3. Stoffel MA, Nakagawa S, Schielzeth H. rptR: Repeatability estimation and variance decomposition by generalized linear mixed-effects models. Methods Ecol Evol. 2017;

4. Terwee CB, Bot SDM, de Boer MR, van der Windt DAWM, Knol DL, Dekker J, et al. Quality criteria were proposed for measurement properties of health status questionnaires. J Clin Epidemiol. 2007;60: 34–42. doi:10.1016/j.jclinepi.2006.03.012
