# Supplementary material for: Postural control in healthy adults: Determinants of trunk sway assessed with a chest-worn accelerometer in 12 quiet standing tasks
Source: PLoS One. 2019 Jan 23;14(1):e0211051. doi: 10.1371/journal.pone.0211051 (PMC6344019; doi:10.1371/journal.pone.0211051)
Supplement: S2 File — The document contains supplementary figures: histograms, scatter plots, and fitted curves of the MARS analysis. (PDF) [file pone.0211051.s002.pdf]

Postural control in healthy adults: determinants of trunk sway assessed with a chest-worn accelerometer in 12 quiet standing tasks

Fabienne Reynard, David Christe, Philippe Terrier

Supplementary Figures

Fig. A Histograms of the sway amplitudes ....p. 2

Fig. B MARS analysis: Feet apart, eyes open, anteroposterior axis .... p. 3
Fig. C MARS analysis: Feet apart, eyes open, mediolateral axis ....p. 4
Fig. D MARS analysis: Feet apart, eyes closed, anteroposterior axis ....p. 5
Fig. E MARS analysis: Feet apart, eyes closed, mediolateral axis .... p. 6
Fig. F MARS analysis: Feet together, eyes open, anteroposterior axis .... p. 7
Fig. G MARS analysis: Feet together, eyes open, mediolateral axis ....p. 8
Fig. H MARS analysis: Feet together, eyes closed, anteroposterior axis ....p. 9
Fig. I MARS analysis: Feet together, eyes closed, mediolateral axis .... p. 10
Fig. J MARS analysis: Feet together on foam, eyes open, anteroposterior axis .... p. 11
Fig. K MARS analysis: Feet together on foam, eyes closed, mediolateral axis .... p. 12
Fig. L MARS analysis: Feet together on foam, eyes open, anteroposterior axis .... .p. 13
Fig. M MARS analysis: Feet together on foam, eyes closed, mediolateral axis .... p. 14
Fig. N MARS analysis: One-leg, average, eyes open, anteroposterior axis ....p. 15
Fig. O MARS analysis: One-leg, average, eyes open, mediolateral axis ....p. 16
Fig. P MARS analysis: One-leg, average, eyes closed, anteroposterior axis................................... p. 17
Fig. Q MARS analysis: One-leg, average, eyes closed, mediolateral axis .... p. 18
Fig. R MARS analysis: One-leg, board, average, eyes open, anteroposterior axis .... p. 19
Fig. S MARS analysis: One-leg, board, average, eyes open, mediolateral axis ....p. 20

Figure Legends S2–S27. Fitting curves were drawn onto the four scatter plots, one for each continuous predictor (age, height, mass, exercise). More precisely, the MARS model was fed with each predictor with others held constant (median). In addition, we predicted the sways separately for males and females to highlight sex effects.

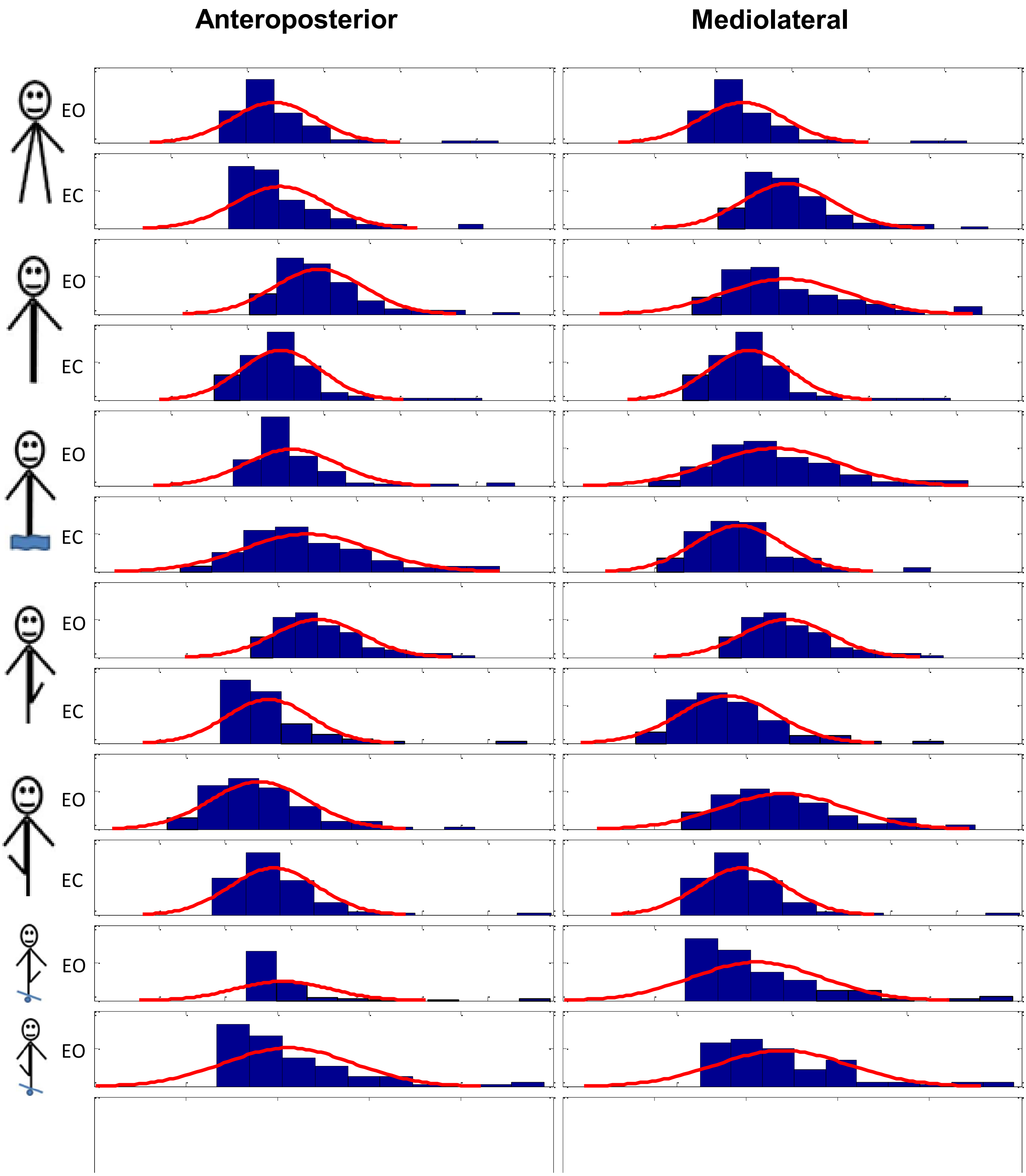

Fig. A Histograms of the sway amplitudes in the 100 participants. The histograms show the distribution of sway amplitude for each standing task (see table 1 in the main document for tasks description). The values were classified in 10 bins and normal fits were added to enhance the interpretation.

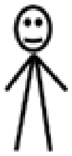

MARS analysis

Figure B

Feet apart, eyes open, anteroposterior axis

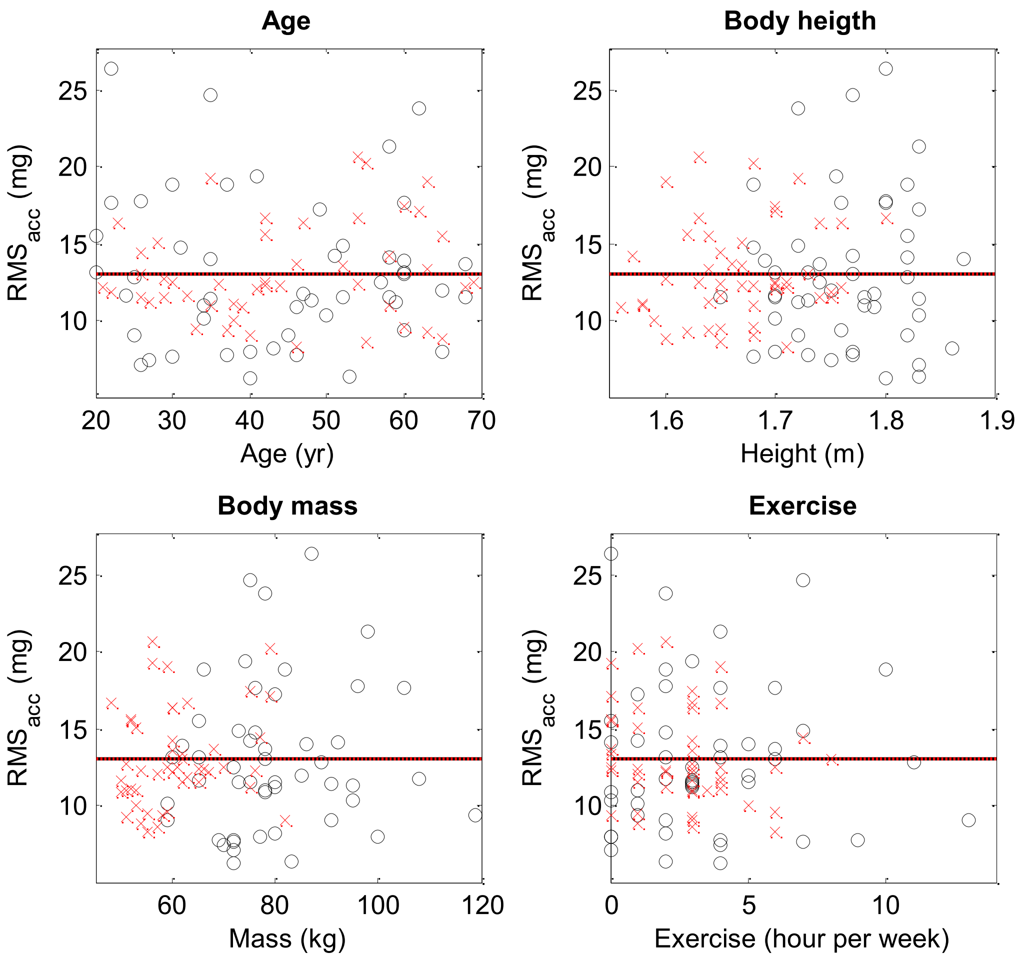

y = 13.069

O : Males
X : Females

Dependent variable:

y: postural sway assessed through RMS acceleration

Predictors in the model:

x1: Age x2: Sex x3: Body height x4: Body mass x5: Exercise

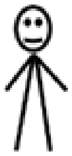

MARS analysis

Figure C

Feet apart, eyes open, mediolateral axis

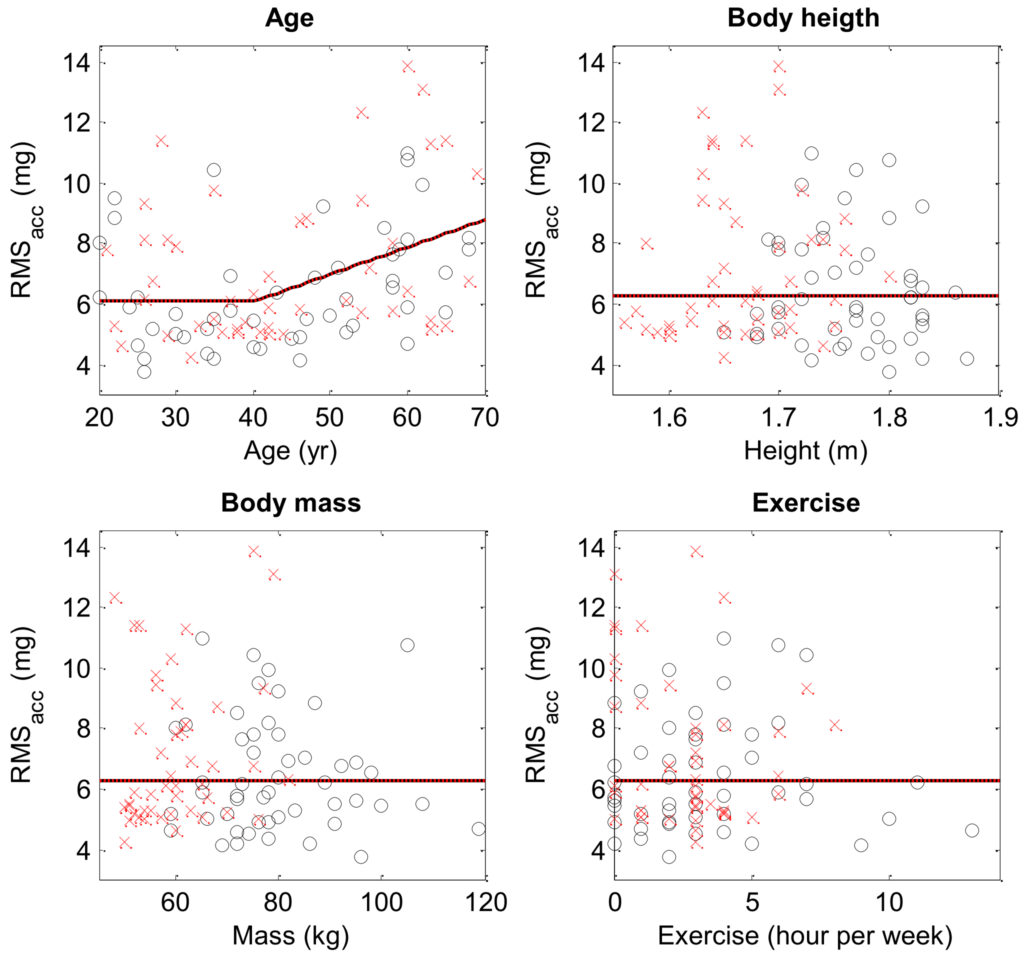

O : Males

X : Females

BF1 = max(0, x1 -40)

y = 6.0873 +0.089716*BF1

Dependent variable:

y: postural sway assessed through RMS acceleration

Predictors in the model:

x1: Age x2: Sex x3: Body height x4: Body mass x5: Exercise

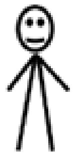

MARS analysis

Figure D

Feet apart, eyes closed, anteroposterior axis

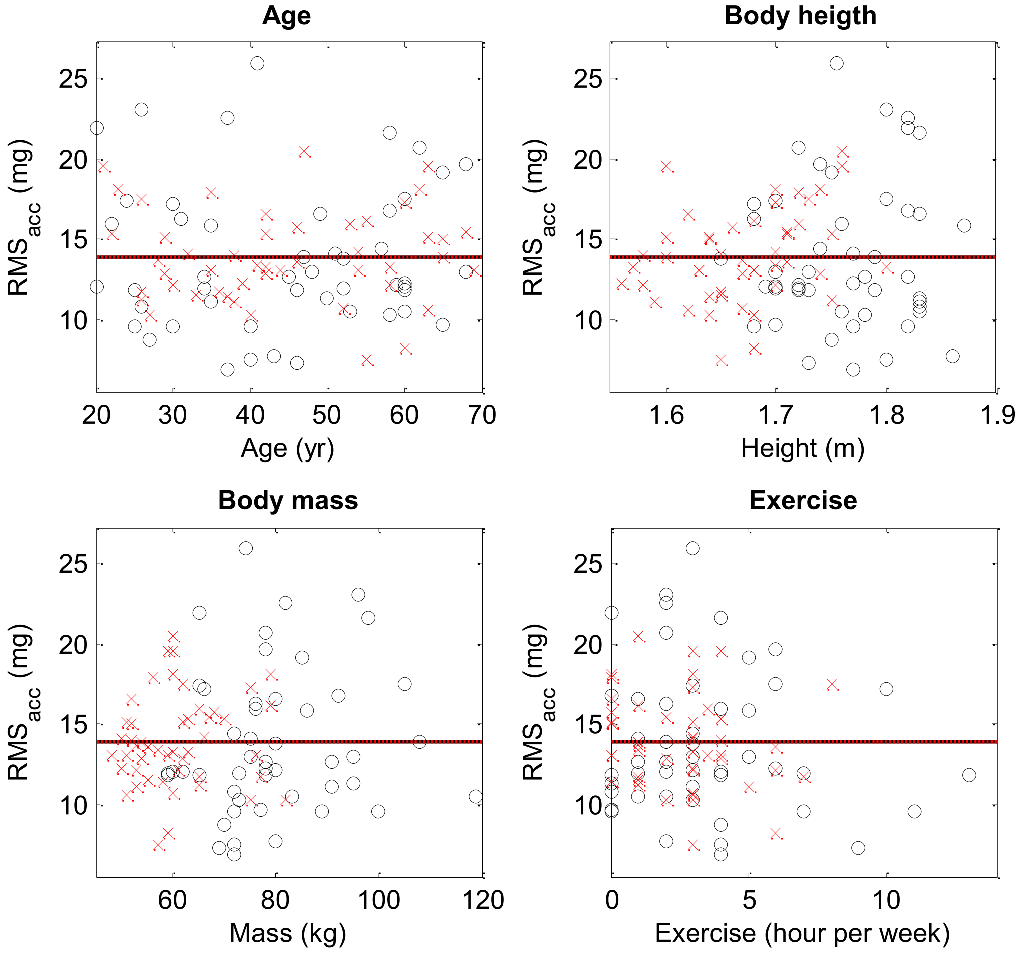

y = 13.882

O : Males
X : Females

Dependent variable:

y: postural sway assessed through RMS acceleration

Predictors in the model:

x1: Age x2: Sex x3: Body height x4: Body mass x5: Exercise

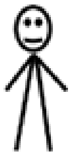

MARS analysis

Figure E

Feet apart, eyes closed, mediolateral axis

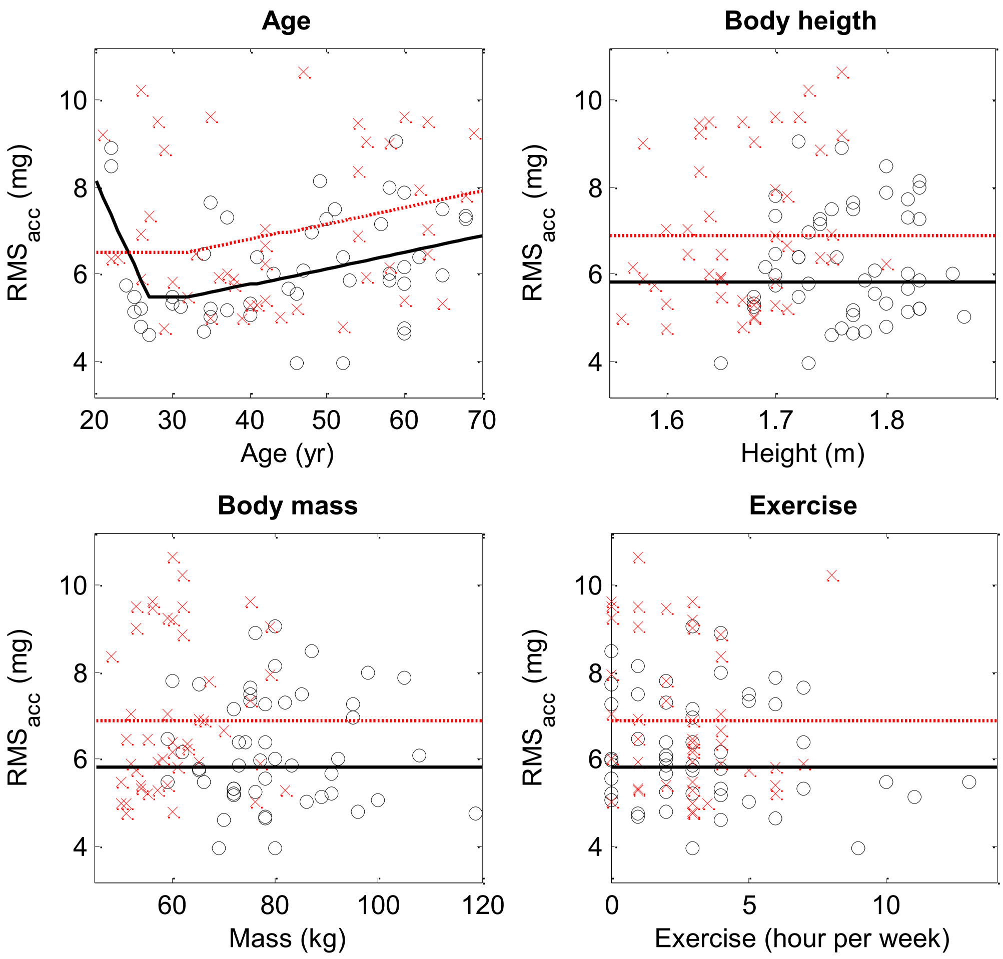

O : Males
X : Females

BF1 = max(0, x1 -32)

BF2 = max(0,1 -x2)

BF3 = BF2 * max(0,27 -x1)

y = 6.4942 +0.037034*BF1 -1.0325*BF2 +0.38002*BF3

Dependent variable:

y: postural sway assessed through RMS acceleration

Predictors in the model:

x1: Age x2: Sex x3: Body height x4: Body mass x5: Exercise

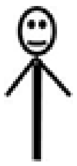

MARS analysis

Figure F

Feet together, eyes open, anteroposterior axis

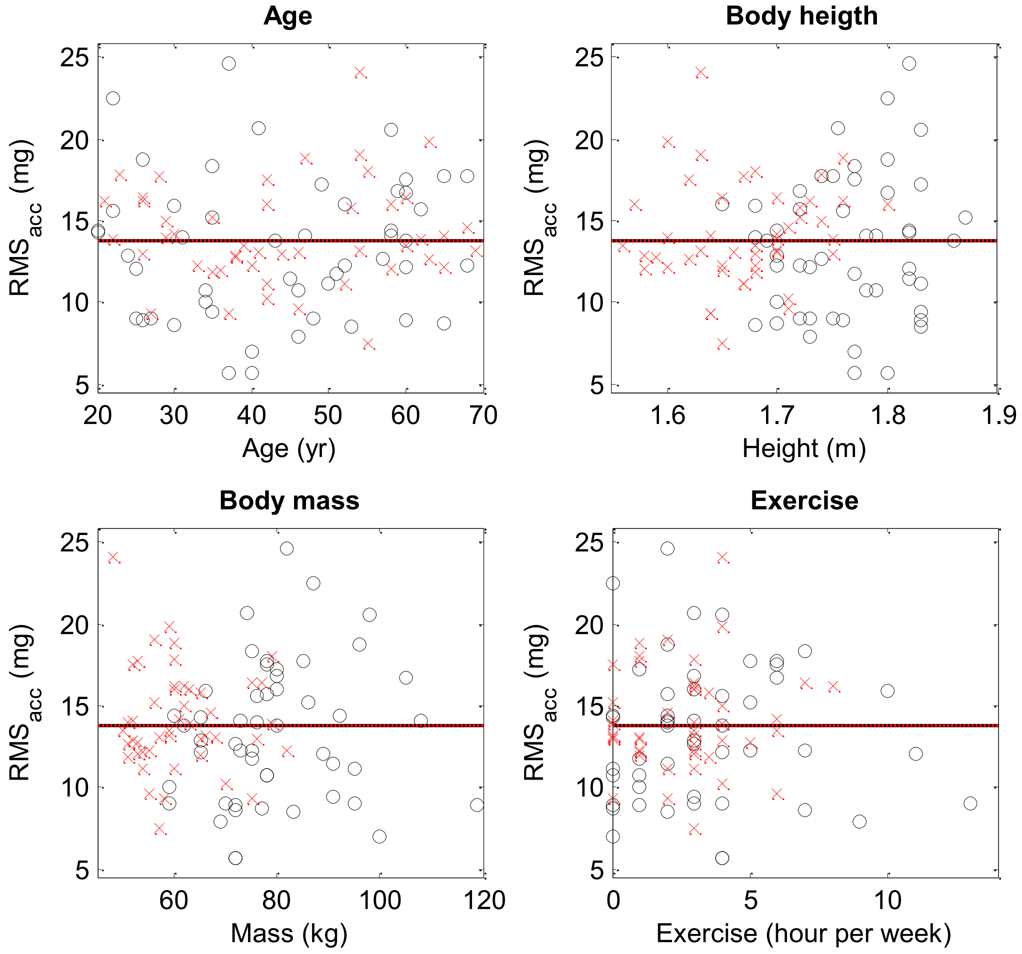

y = 13.749

O : Males
X : Females

Dependent variable:

y: postural sway assessed through RMS acceleration

Predictors in the model:

x1: Age x2: Sex x3: Body height x4: Body mass x5: Exercise

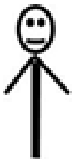

MARS analysis

Figure G

Feet together, eyes open, mediolateral axis

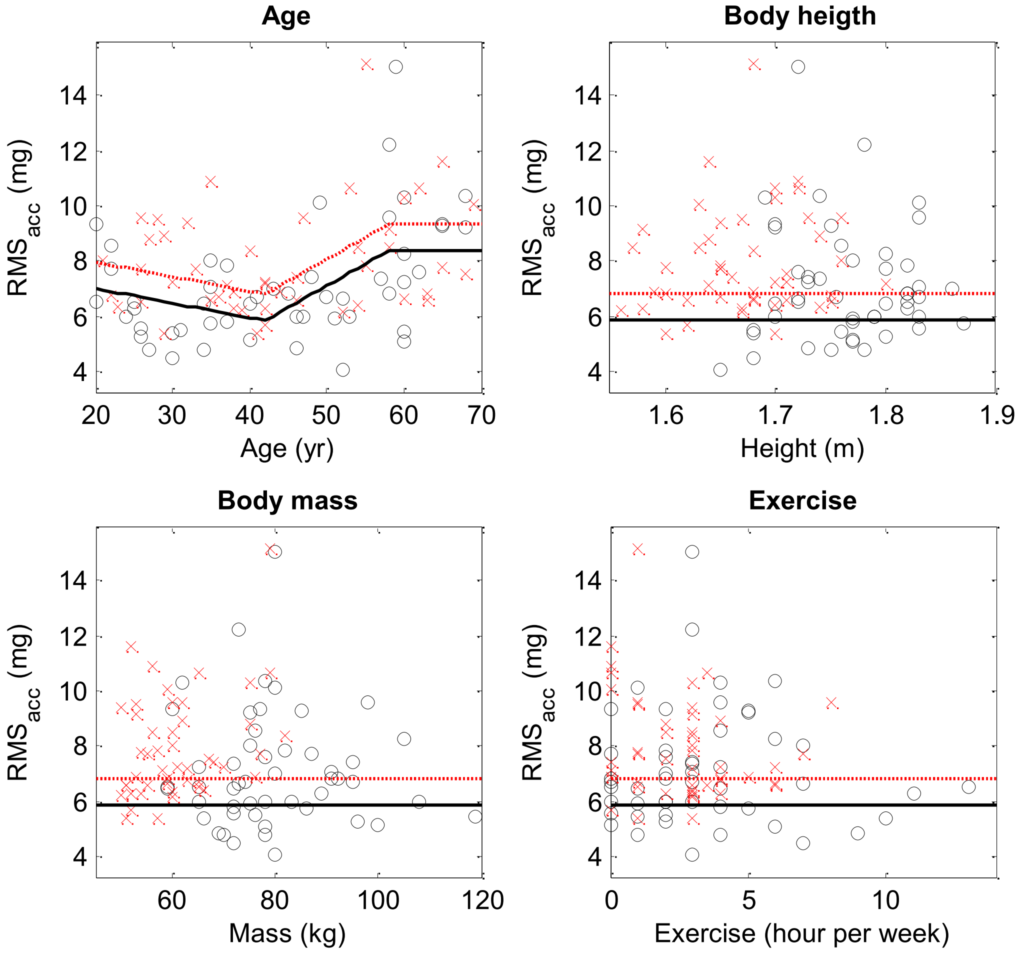

O : Males
X : Females

BF1 = max(0,42 -x1)

BF2 = max(0,1 -x2)

BF3 = max(0,58 -x1)

y = 9.344 +0.21206*BF1 -0.96173*BF2 -0.15885*BF3

Dependent variable:

y: postural sway assessed through RMS acceleration

Predictors in the model:

x1: Age   x2: Sex   x3: Body height   x4: Body mass   x5: Exercise

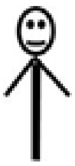

MARS analysis

Figure H

Feet together, eyes closed, anteroposterior axis

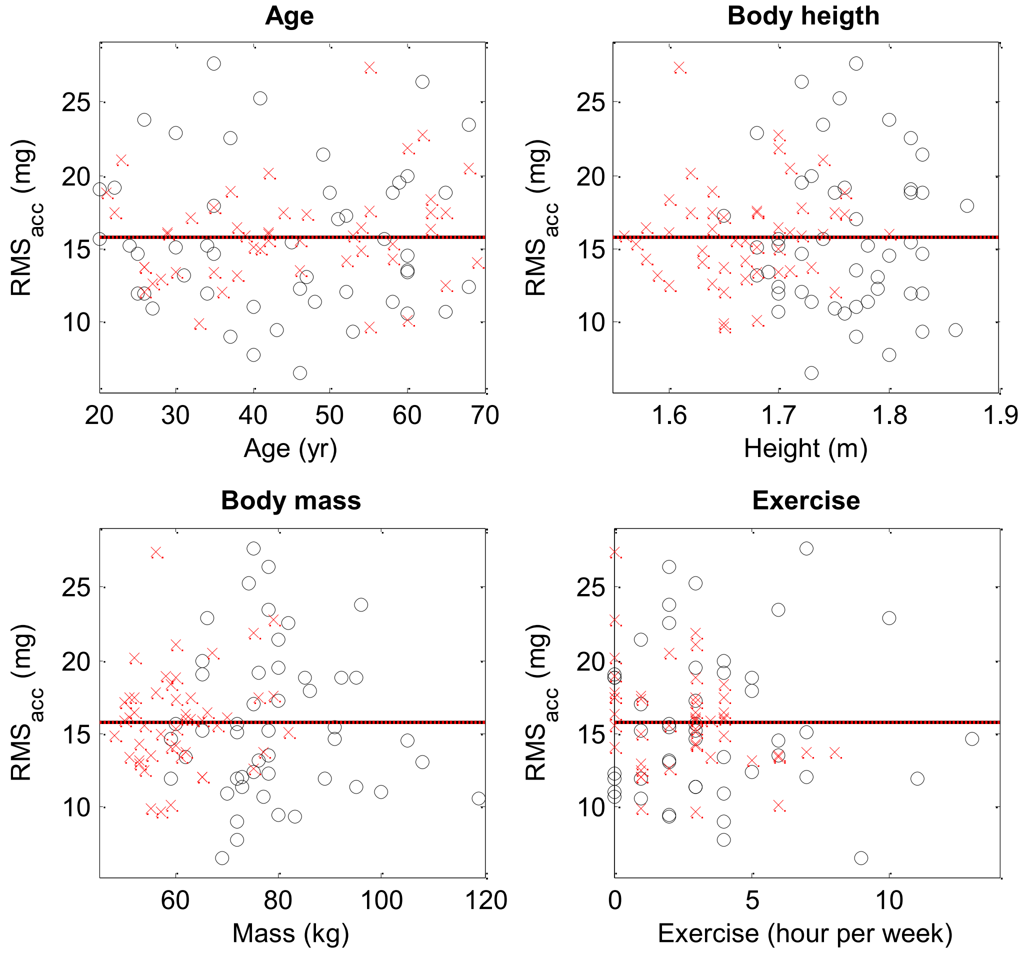

y = 15.809

O : Males
X : Females

Dependent variable:

y: postural sway assessed through RMS acceleration

Predictors in the model:

x1: Age x2: Sex x3: Body height x4: Body mass x5: Exercise

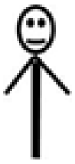

MARS analysis

Figure I

Feet together, eyes closed, mediolateral axis

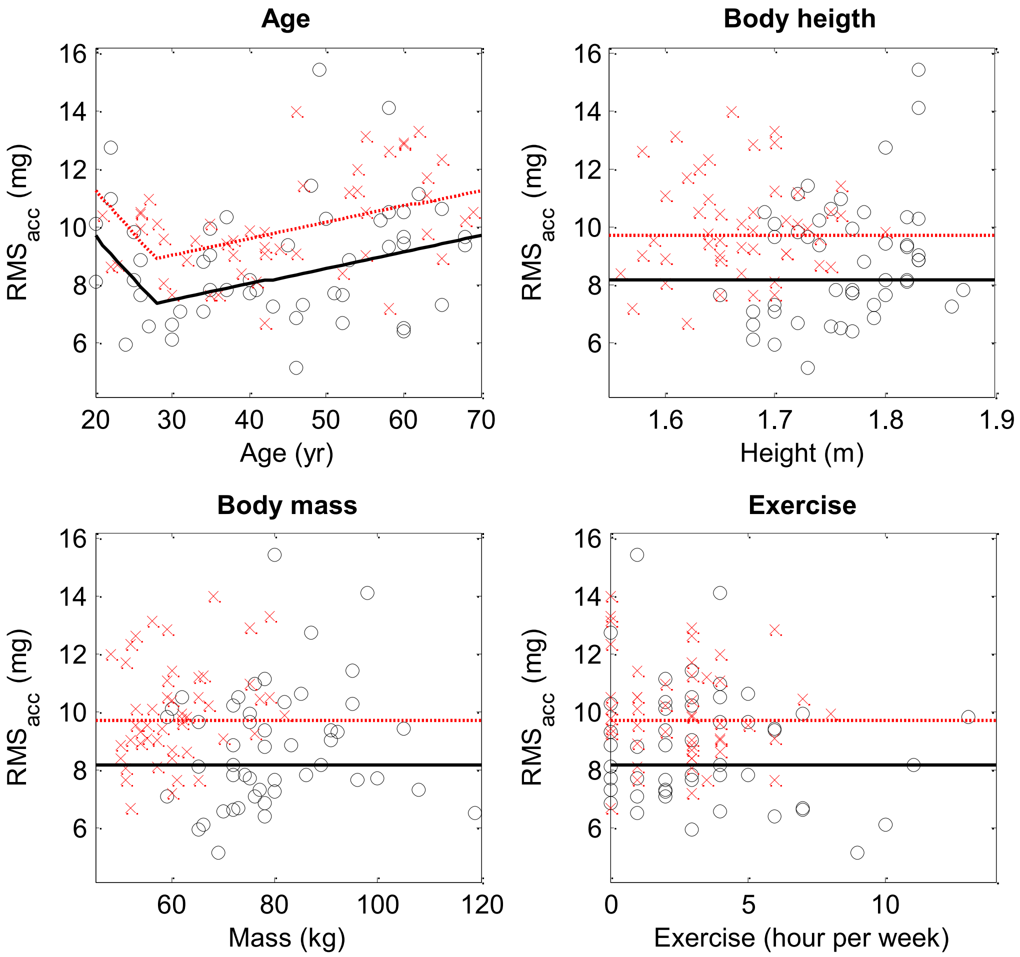

O : Males
X : Females

BF1 = max(0, x1 -28)

BF2 = max(0,28 -x1)

BF3 = max(0, x2 +0)

BF4 = max(0, x3 -1.77) * max(0, x1 -43)

y = 7.3414 +0.056253*BF1 +0.2924*BF2 +1.574*BF3 +3.5727*BF4

Dependent variable:

y: postural sway assessed through RMS acceleration

Predictors in the model:

x1: Age x2: Sex x3: Body height x4: Body mass x5: Exercise

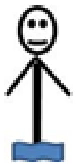

MARS analysis

Figure J

Feet together on foam, eyes open, anteroposterior axis

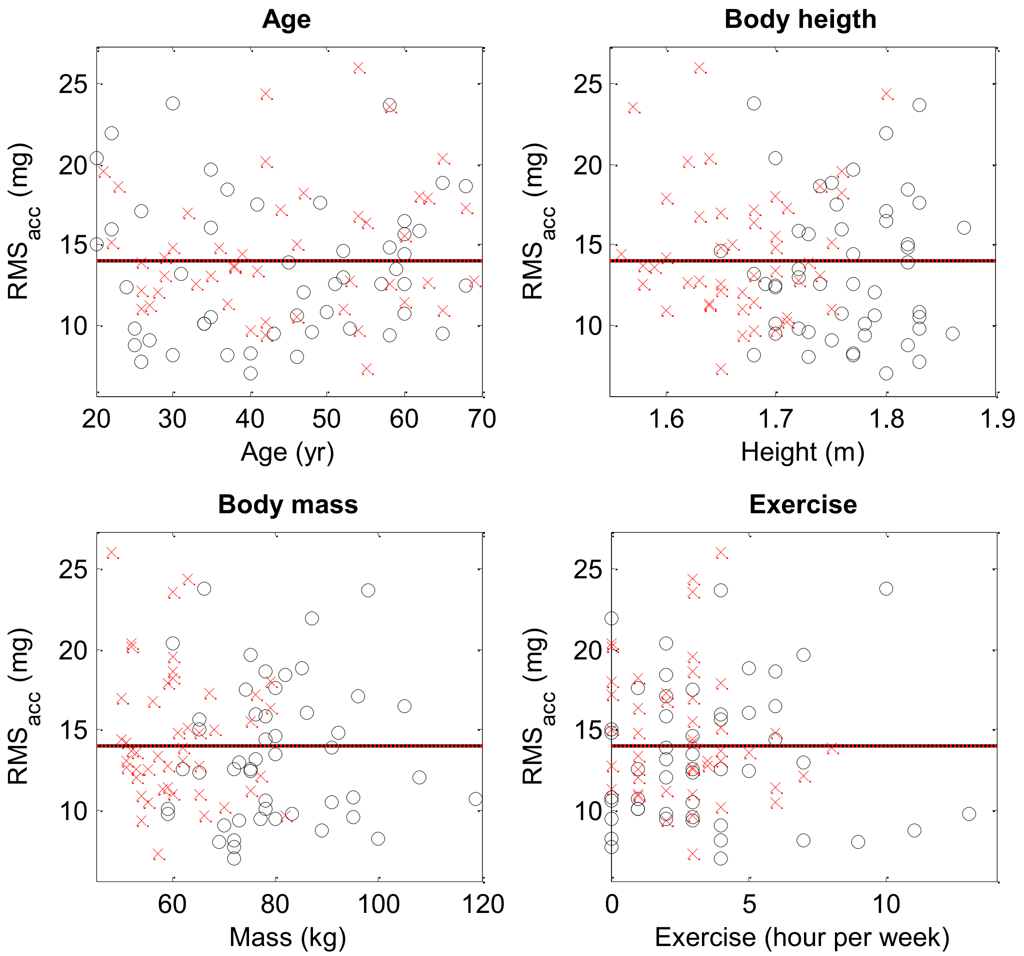

y = 13.983

O : Males

X : Females

Dependent variable:

y: postural sway assessed through RMS acceleration

Predictors in the model:

x1: Age x2: Sex x3: Body height x4: Body mass x5: Exercise

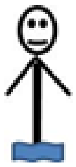

MARS analysis

Figure K

Feet together on foam, eyes open, mediolateral axis

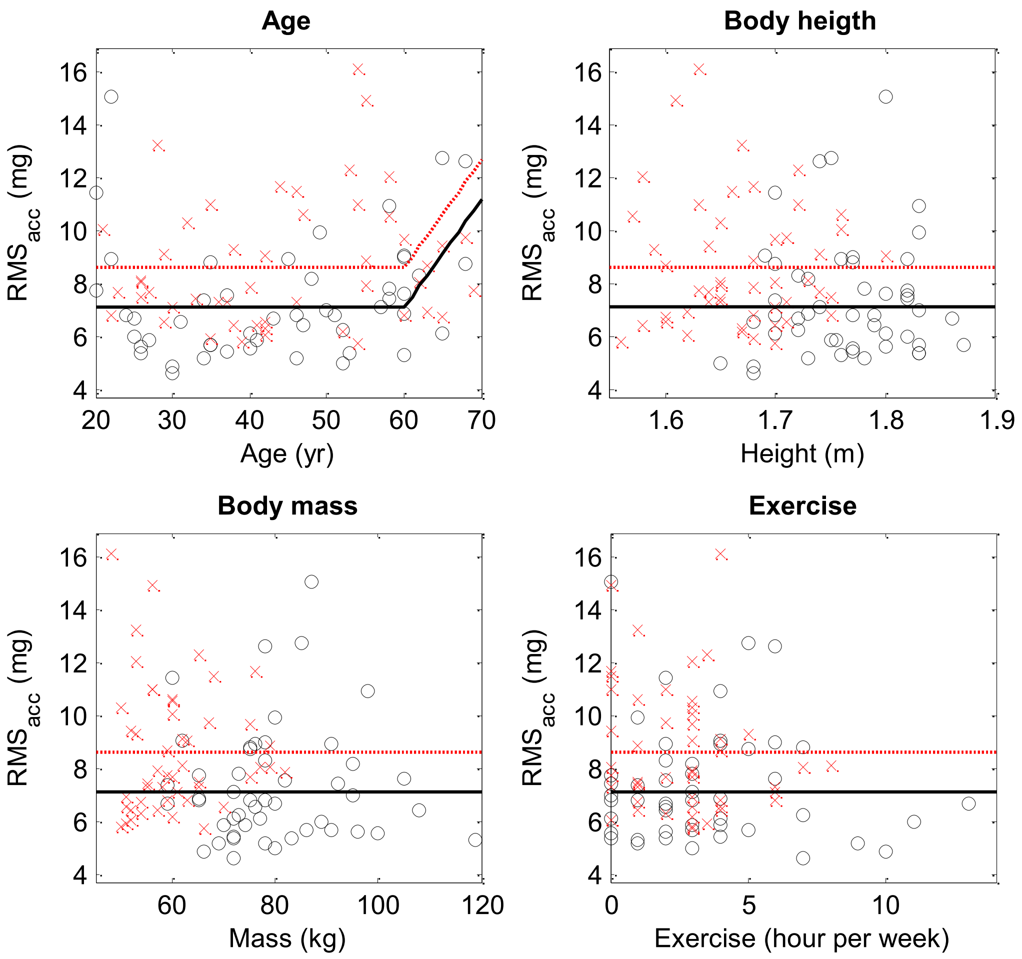

O : Males
X : Females

BF1 = max(0, x2 +0)

BF2 = max(0, x3 -1.67) * max(0, x1 -60)

y = 7.0942 +1.5104*BF1 +8.9817*BF2

Dependent variable:

y: postural sway assessed through RMS acceleration

Predictors in the model:

x1: Age x2: Sex x3: Body height x4: Body mass x5: Exercise

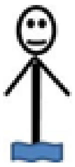

MARS analysis

Figure L

Feet together on foam, eyes closed, anteroposterior axis

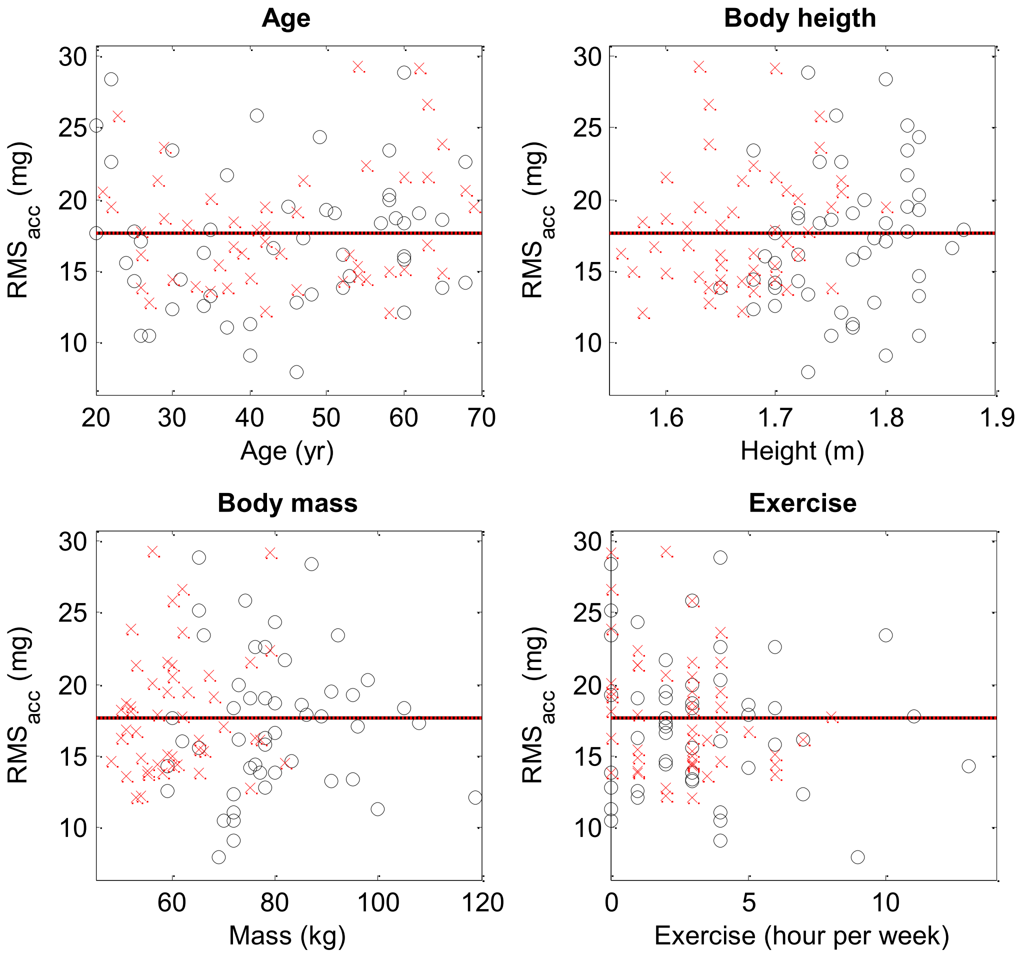

y = 17.617

O : Males
X : Females

Dependent variable:

y: postural sway assessed through RMS acceleration

Predictors in the model:

x1: Age x2: Sex x3: Body height x4: Body mass x5: Exercise

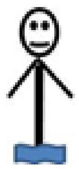

MARS analysis

Figure M

Feet together on foam, eyes closed, mediolateral axis

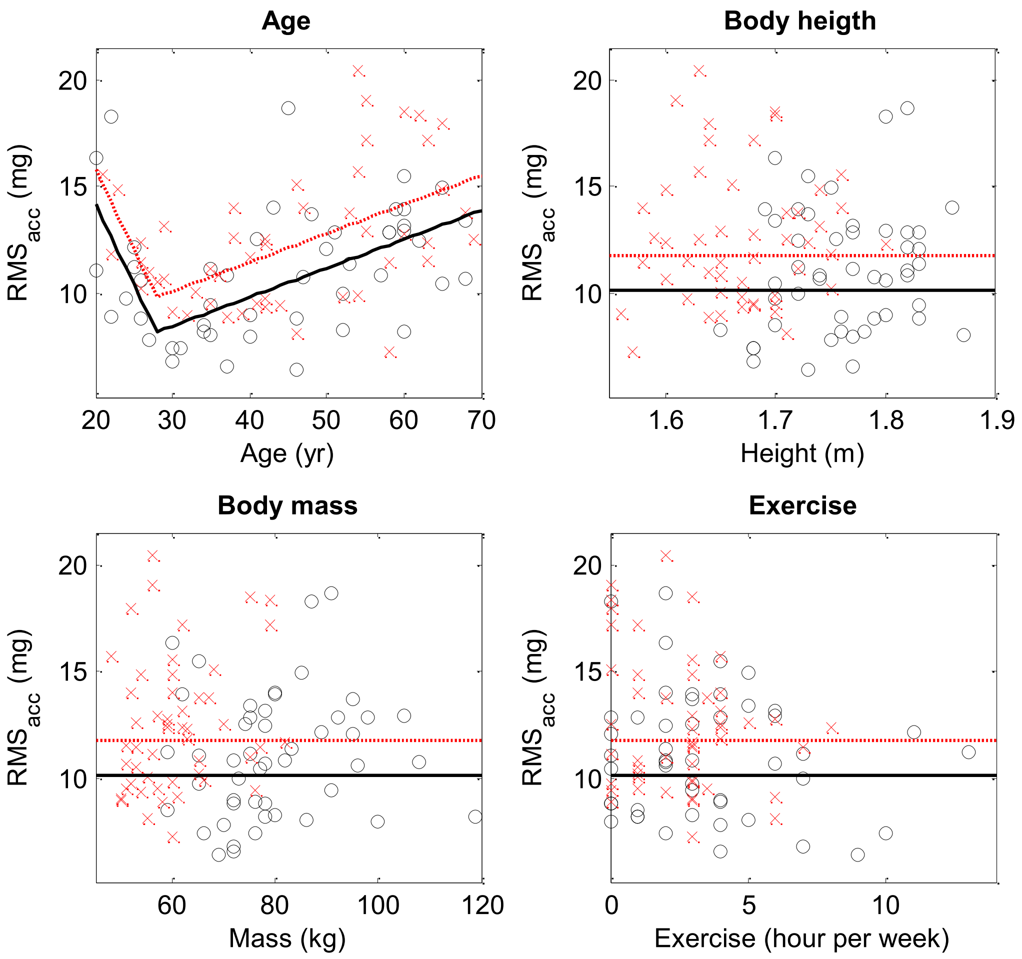

O : Males
X : Females

BF1 = max(0, x1 -28)

BF2 = max(0,28 -x1)

BF3 = max(0, x2 +0)

y = 8.2342 +0.13476*BF1 +0.74456*BF2 +1.6215*BF3

Dependent variable:

y: postural sway assessed through RMS acceleration

Predictors in the model:

x1: Age x2: Sex x3: Body height x4: Body mass x5: Exercise

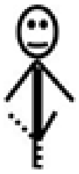

MARS analysis

Figure N

One-leg, average, eyes open, anteroposterior axis

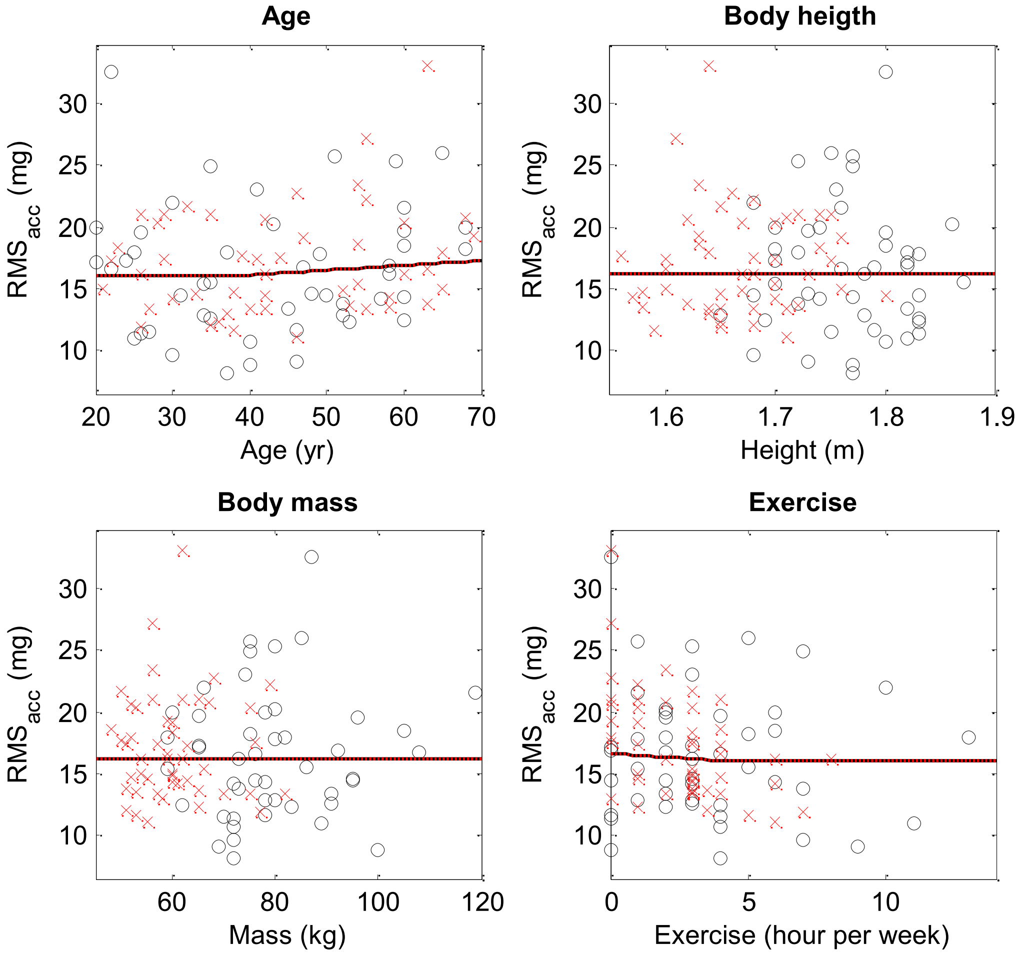

O : Males
X : Females

BF1 = max(0, x1 -40) * max(0, 3.5 -x5)

y = 16.111 +0.076946*BF1

Dependent variable:

y: postural sway assessed through RMS acceleration

Predictors in the model:

x1: Age x2: Sex x3: Body height x4: Body mass x5: Exercise

MARS analysis

Figure O

One-leg, average, eyes open, mediolateral axis

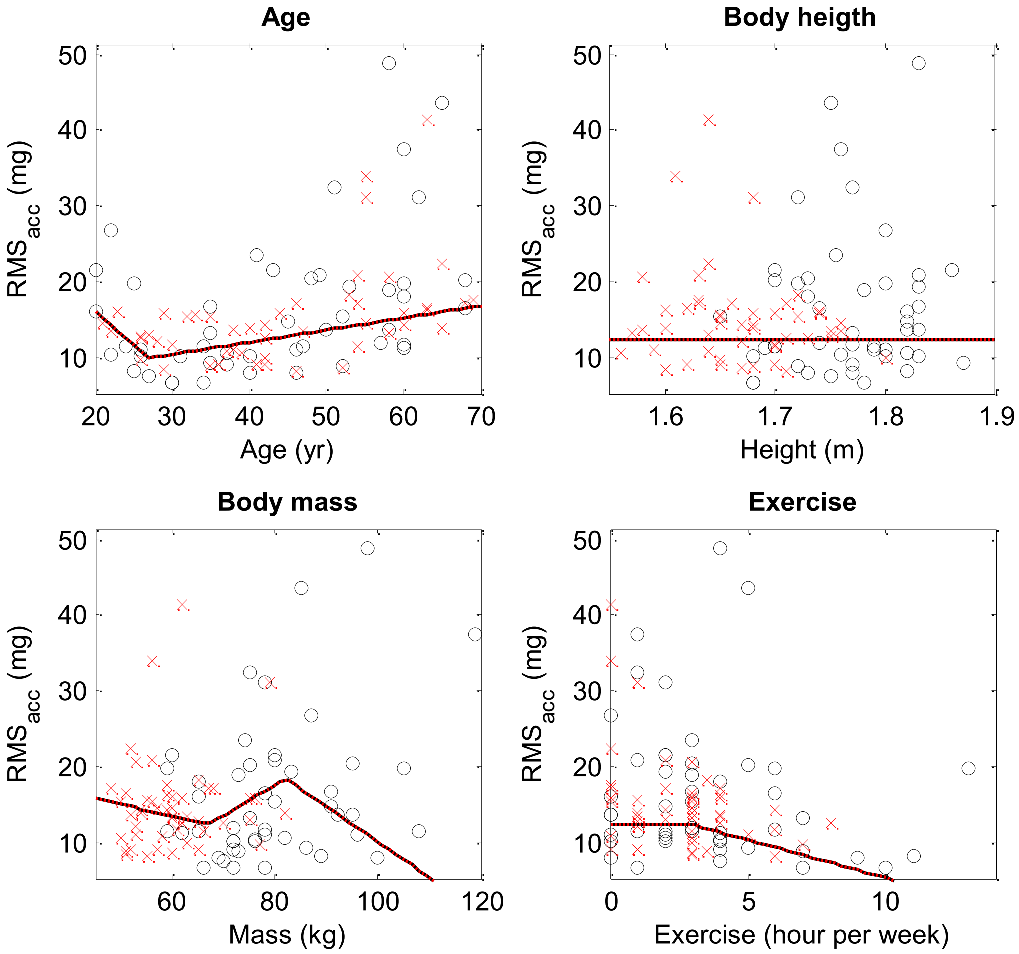

O : Males
X : Females

$$BF1 = \max(0, x1 - 27) * \max(0, x4 - 67)$$

$$BF2 = \max(0, x1 - 27) * \max(0, x5 - 3.5)$$

$$BF3 = \max(0, x4 - 82)$$

$$BF4 = BF3 * \max(0, 35 - x1)$$

$$BF5 = \max(0, 82 - x4) * \max(0, x1 - 27)$$

$$BF6 = \max(0, 82 - x4) * \max(0, 27 - x1)$$

$$y = 10.05 + 0.037578*BF1 - 0.066242*BF2 - 1.0277*BF3 + 0.15092*BF4 + 0.010644*BF5 + 0.05885*BF6$$

Dependent variable:

y: postural sway assessed through RMS acceleration

Predictors in the model:

x1: Age x2: Sex x3: Body height x4: Body mass x5: Exercise

MARS analysis

Figure P

One-leg, average, eyes closed, anteroposterior axis

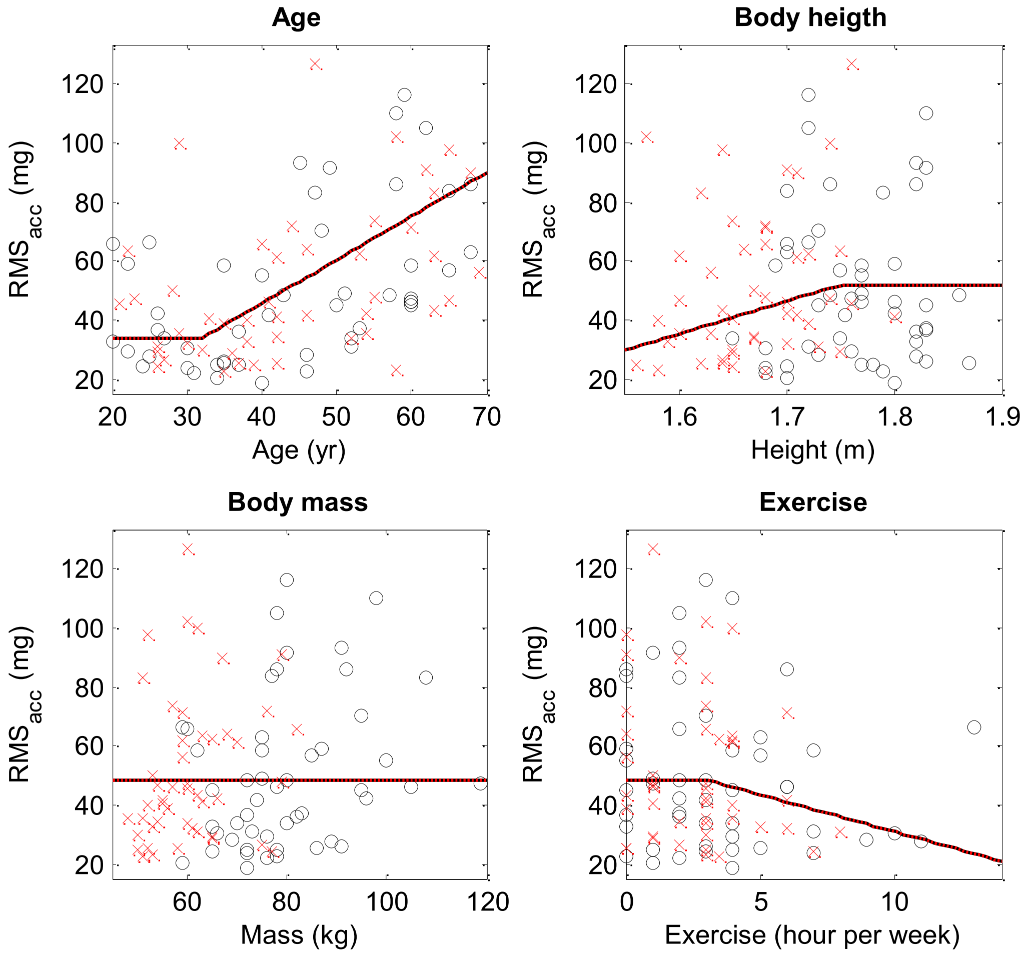

BF1 = max(0, x1 -32)

BF2 = BF1 * max(0, x5 -3)

BF3 = max(0,1.75 -x3)

BF4 = max(0,65 -x4) * max(0,32 -x1)

y = 37.369 +1.4676*BF1 -0.25038*BF2 -110.2*BF3 +0.4598*BF4

Dependent variable:

y: postural sway assessed through RMS acceleration

Predictors in the model:

x1: Age x2: Sex x3: Body height x4: Body mass x5: Exercise

MARS analysis

Figure Q

One-leg, average, eyes closed, mediolateral axis

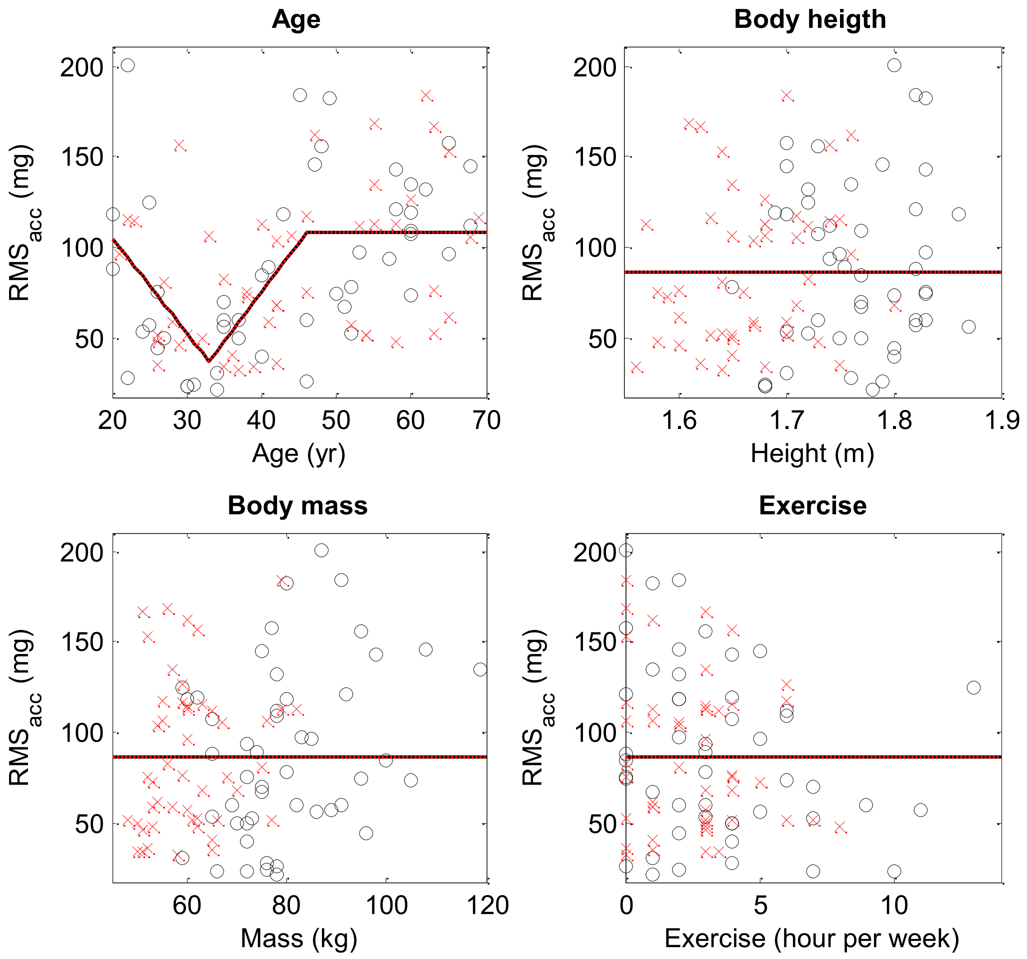

O : Males
X : Females

BF1 = max(0,33 -x1)

BF2 = max(0,46 -x1)

y = 108.31 +10.733*BF1 -5.4939*BF2

Dependent variable:

y: postural sway assessed through RMS acceleration

Predictors in the model:

x1: Age x2: Sex x3: Body height x4: Body mass x5: Exercise

MARS analysis

Figure R

One-leg, board, average, eyes open anteroposterior axis

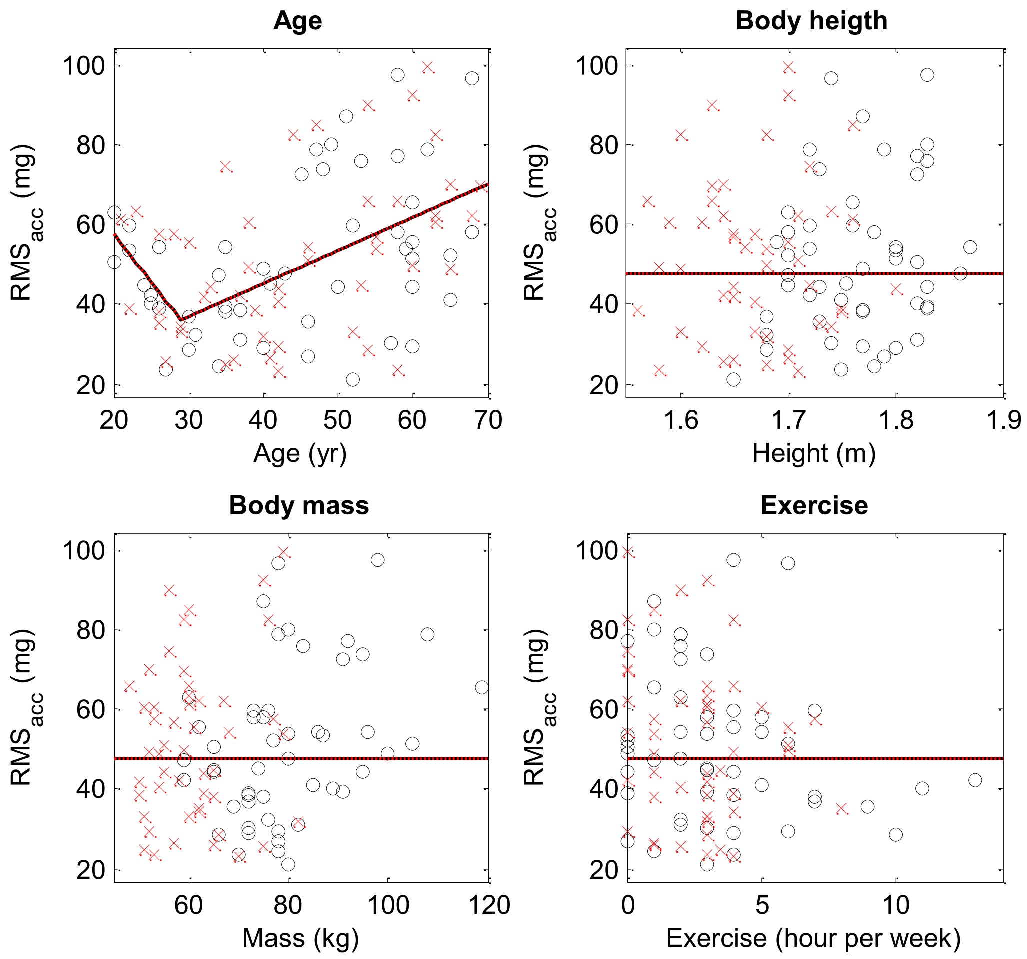

O : Males
X : Females

BF1 = max(0, x1 -29)

BF2 = max(0,29 -x1)

y = 36.046 +0.82999*BF1 +2.3834*BF2

Dependent variable:

y: postural sway assessed through RMS acceleration

Predictors in the model:

x1: Age x2: Sex x3: Body height x4: Body mass x5: Exercise

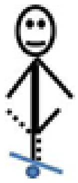

MARS analysis

Figure S

One-leg, board, average, eyes open mediolateral axis

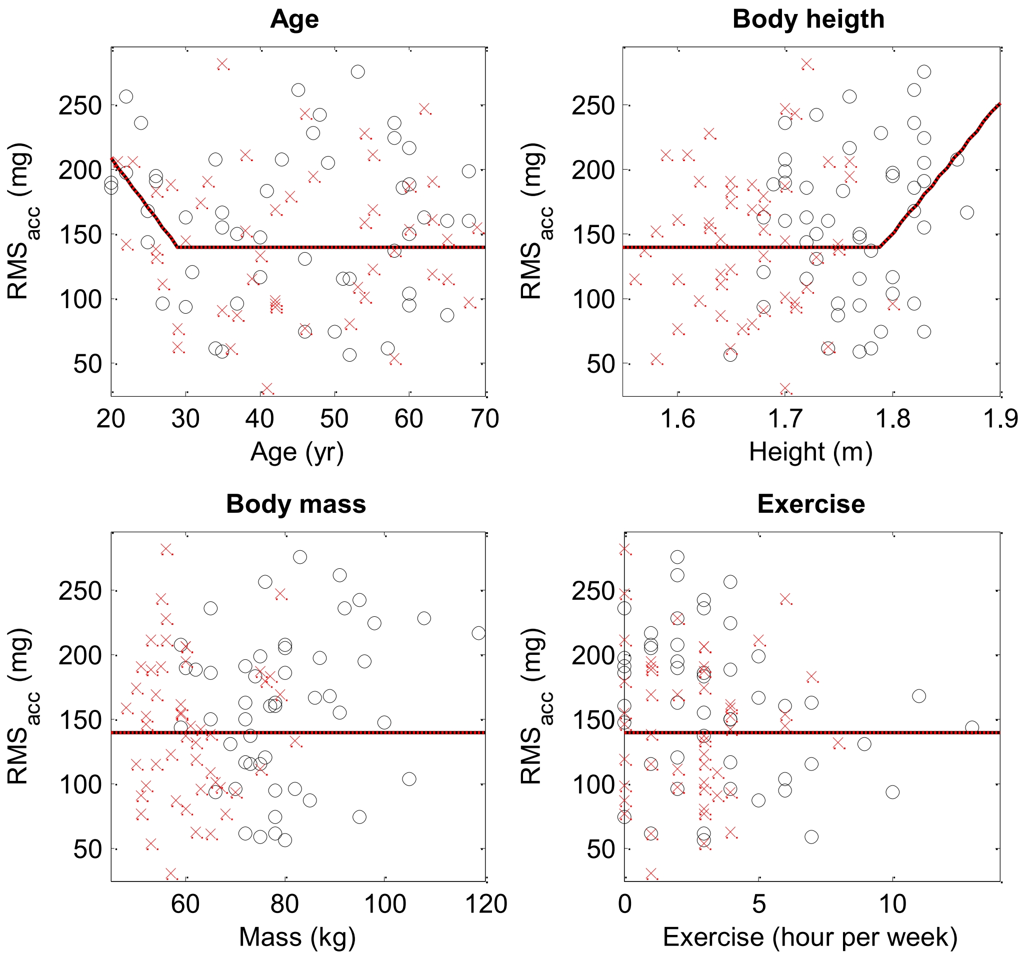

O : Males
X : Females

BF1 = max(0,29 -x1)

BF2 = max(0, x1 -29) * max(0, x3 -1.79)

y = 139.65 +7.6657*BF1 +70.053*BF2

Dependent variable:

y: postural sway assessed through RMS acceleration

Predictors in the model:

x1: Age x2: Sex x3: Body height x4: Body mass x5: Exercise
